# Mathematical Modeling of Ion Quantum Tunneling Reveals Novel Properties of Voltage-Gated Channels and Quantum Aspects of Their Pathophysiology in Excitability-Related Disorders

**DOI:** 10.3390/pathophysiology28010010

**Published:** 2021-03-07

**Authors:** Abdallah Barjas Qaswal, Omar Ababneh, Lubna Khreesha, Abdallah Al-Ani, Ahmad Suleihat, Mutaz Abbad

**Affiliations:** 1 Department of Internship Program, Jordan University Hospital, The University of Jordan, Amman 11942, Jordan; 2 Department of Anesthesia and Intensive Care, School of Medicine, The University of Jordan, Amman 11942, Jordan; omar.ababneh@ju.edu.jo; 3 Department of Special Surgery, School of Medicine, The University of Jordan, Amman 11942, Jordan; l.khreesha@ju.edu.jo; 4 School of Medicine, The University of Jordan, Amman 11942, Jordan; abdallahalany@gmail.com; 5 Department of General Surgery, School of Medicine, The University of Jordan, Amman 11942, Jordan; Ahmad_slaihat@yahoo.com (A.S.); Mo3taz.3bbad@gmail.com (M.A.)

**Keywords:** quantum tunneling, voltage-gated channel, sodium ions, potassium ions, quantum biophysics, epilepsy, arrhythmias, pain, quantum biology

## Abstract

Voltage-gated channels are crucial in action potential initiation and propagation and there are many diseases and disorders related to them. Additionally, the classical mechanics are the main mechanics used to describe the function of the voltage-gated channels and their related abnormalities. However, the quantum mechanics should be considered to unravel new aspects in the voltage-gated channels and resolve the problems and challenges that classical mechanics cannot solve. In the present study, the aim is to mathematically show that quantum mechanics can exhibit a powerful tendency to unveil novel electrical features in voltage-gated channels and be used as a promising tool to solve the problems and challenges in the pathophysiology of excitability-related diseases. The model of quantum tunneling of ions through the intracellular hydrophobic gate is used to evaluate the influence of membrane potential and gating free energy on the tunneling probability, single channel conductance, and quantum membrane conductance. This evaluation is mainly based on graphing the mathematical relationships between these variables. The obtained mathematical graphs showed that ions can achieve significant quantum membrane conductance, which can affect the resting membrane potential and the excitability of cells. In the present work, quantum mechanics reveals original electrical properties associated with voltage-gated channels and introduces new insights and implications into the pathophysiology of excitability- related disorders. In addition, the present work sets a mathematical and theoretical framework that can be utilized to conduct experimental studies in order to explore the quantum aspects of voltage-gated channels and the quantum bioelectrical property of biological membranes.

## 1. Introduction

Voltage-gated channels are crucial for action potential initiation and propagation [1]. Thus, any disturbance in their function or structure could affect the processes and actions within, and of excitable cells, resulting in different diseases, such as epilepsy [2], pain disorders [3], and cardiac arrhythmias [4]. Additionally, understanding how voltage-gated channels operate and how they can be implicated in the pathophysiology of many diseases is based mainly on the principles of classical mechanics and laws of thermodynamics [1]. Such practice ignores the role of quantum mechanics as its integration within classical mechanics might unravel new aspects regarding the function of voltage-gated channels, which could enhance our understanding of excitable cells and their role in the pathogenesis of some diseases. Furthermore, the integration of quantum mechanics is further encouraged by the many puzzling challenges associated with understanding the roles of abnormal voltage-gated channels in the pathophysiology of excitability-related disorders such as epilepsy [2,5], pain disorders [6], and cardiac arrhythmias [7]. Those diseases represent major medical issues and hurdles in terms of how they are prevented, treated, or controlled [8,9,10,11].

Quantum mechanics is the field of physics that focuses on the behavior of atomic and subatomic particles and this behavior can be studied by using the Schrödinger equation to obtain the wave function of a particle [12]. Recently, the field of quantum mechanics has been extended to biology in order to understand different biological actions and events including photosynthesis, action of enzymes, olfaction, and birds’ navigation [13,14,15]. However, many concerns have emerged along quantum biology. One of these concerns is that the hot noisy biological environment does not sustain quantum behavior. This concern has been opposed by recent research, which showed that quantum properties, such as the quantum entanglement of huge number of atoms, can be maintained at high temperatures such as those of the human body or even higher [16]. Different mechanisms could explain the persistence of quantum coherence within biological systems, including hydrophobic pockets, as in the hydrophobic gate of the voltage-gated channels that are the focus of this study [17,18,19]. Therefore, this gives researchers more motivation to pursue applying quantum mechanics within biological systems. In recent years, researchers have been focusing on the quantum features of ions, such as potassium and calcium ions, and their role in the processes and actions of neurons [20,21,22]. This approach is scientifically sound as it has been observed and documented that atoms, ions, and even molecules can behave according to the principles of quantum mechanics in a similar fashion to that of subatomic particles [23,24,25,26]. Moreover, the ability of scientists to explain the high selectivity of voltage-gated channels through mathematical structures acts as further evidence for the validity of studying biological systems through a quantum perspective [22,27,28,29]. However, a principal component of the voltage-gated channel did not receive enough attention. This component is the channel’s gate, which determines the channel’s conductance and consequently, the overall membrane’s conductance and the electrical properties of its associated tissues [1,18,19,30]. Thus, the present work is an extension of the previous works that focused on studying the gates of voltage-gated channels through understanding the quantum behavior of their target ions, and their quantum conductance [31,32]. 

This study aims to approach the function of voltage-gated channels from a quantum perspective using the quantum tunneling model [31,32]. The model is designed to portray the novel electrical features of voltage-gated channels and shed light on the differences that make it distinct from the classical model of Boltzmann distribution for voltage-gated channels. Moreover, this quantum model is implemented to signify the contribution of quantum behavior of ions in the pathophysiology of excitable tissue diseases. In this work, the aim is not to focus on and review the details of the challenges and puzzles in the function of voltage-gated channels and their related diseases specifically. However, the aim is to spot hints and clues and to establish a comprehensive mathematical model to encourage researchers to consider quantum mechanics in future works when they aim to resolve a challenge or a puzzle in the field of electrophysiology and to unveil the pathogenesis of a certain disease related to the function of voltage-gated channels. However, some of the challenges and puzzles will be discussed in this work and the quantum model will offer reasonable explanation for them especially that they are not well explained by the classical models. Additionally, the present study might be helpful to aid in developing novel agents to treat and control epilepsy syndromes, pain disorders, and cardiac arrhythmias.

## 2. The Mathematical Model

### 2.1. The Conductance of the Voltage-Gated Channels According to the Laws of Thermodynamics

The voltage-gated channels are mainly composed from two parts: (1) A selectivity filter and (2) an intracellular gate [1]. The function of the selectivity filter is to discriminate between ions and to enable the channel to selectively pass specific ion [1]. On the other hand, the intracellular gate functions as the main controller of the channel’s conductance and that of the overall membrane [18,19,30]. The intracellular gate is a hydrophobic constriction made by the bundles crossing of the four S6 segments of the alpha subunits of the channel [18,19]. This gate represents an energy barrier that impends the passage of ions [30]. Hence, the intracellular hydrophobic gate controls the ions’ passage and the channel’s conductance. Furthermore, the intracellular gate operates as a narrow hydrophobic pore, in which its ‘open’ state is characterized by an increased pore radius and a decreased energy barrier, which consequently facilities the passage of ions [33]. According to the classical physics of thermodynamics, the voltage gated channels have two states: (1) A closed state (C) and (2) an open state (O), which fit the Boltzmann distribution, as demonstrated in the following equation [1,33]:

(1) $$P=\frac{O}{C+O}={\left(1+e^{\frac{q_{g}V_{1/2}-q_{g}V_{m}}{K_{B}T}}\right)}^{-1},$$

where *P* is the fraction of open channels from the total available channels at a certain area of the cell membrane (the open probability), *V_1/2_* is the membrane voltage at which half of the channels are open, *V_m_* is the actual membrane voltage, *q_g_* is the gating’s charge, *K_B_* is the Boltzmann’s constant ($1.38\times 10^{-23}$ J/K), and *T* is the absolute body temperature (310 K). The mathematical term $q_{g}V_{1/2}$ represents the gating free energy, which is the energy associated with the conversion from the ‘closed’ state to the ‘open’ state at $V_{m}=0$ [34]. Furthermore, the mathematical structure $q_{g}V_{1/2}-q_{g}V_{m}$ represents the energy required to switch from the ‘closed’ state to the ‘open’ state or the energy barrier that resists the passage of ions at a certain membrane potential *V_m_*. 

According to the Boltzmann distribution, voltage-gated channels are either in the ‘closed’ state that has zero conductance, or in the ‘open’ state that has a certain value of conductance $C_{\sin gle}$. As a result, when there is a certain number of channels at a surface area of a membrane with a certain membrane voltage *V_m_*, a fraction of this number of channels will be ‘open’ and be able to conduct ions. Therefore, the membrane conductance *C_M_* due to voltage-gated channels can be calculated by the following equation:

(2) $$C_{M}=D\times C_{\sin gle}\times {\left(1+e^{\frac{q_{g}V_{1/2}-q_{g}V_{m}}{K_{B}T}}\right)}^{-1},$$

where *C_M_* is the membrane’s conductance (S/m^2^), $C_{\sin gle}$ is the single channel conductance (S), and *D* is the channels’ density (channels/m^2^). 

### 2.2. The Conductance of the Voltage-Gated Channels According to Quantum Mechanics 

According to quantum mechanics, the hydrophobic gate can be represented as a potential barrier through which ions can tunnel [31,32]. The tunneling probability (*T_Q_*) through the hydrophobic gate, as solved from Schrodinger’s equation, can be calculated by using the following equation [12,31,32]:

(3) $$T_{Q}=e^{\frac{-\sqrt{8m}}{\hbar }\int_{X1}^{X2}\sqrt{(U(x)-KE}dx},$$

where *m* is the mass of the ion, ℏ is the reduced Planck’s constant ($1.05\times 10^{-34}$ Js), *U(x)* is the barrier’s energy with respect to the ion’s position *x* across the gate, *KE* is the kinetic energy of the ion, and *x_1_-x_2_* is the forbidden region of the gate where the barrier’s energy *U(x)* is higher than the kinetic energy of the ion *KE*. 

The energy required to open the gate is ($q_{g}V_{1/2}-q_{g}V_{m}$), which represents the energy barrier of the gate. In other words, this energy is needed to perform the mechanical work to dilate the hydrophobic pore in order to facilitate ion conduction [33]. As a result, we can deduce that ion must have this amount of energy to overcome the energy barrier of the gate. Therefore, this amount of energy ($q_{g}V_{1/2}-q_{g}V_{m}$) represents the energy barrier *U(x)* stated in Equation (3). However, quantum tunneling of ions does not require the dilation of the hydrophobic gate.

Here, it is claimed that this energy distributes equally along the length of the gate *L*. To make a mathematical connection between the barrier’s energy *U(x)* and the ion’s position *x* through the gate, the gate can be illustrated as an electric field that opposes the passage of ions. This electric field *E* can be calculated as demonstrated in the following equation [31,32]:

(4) $$E=\frac{q_{g}V_{1/2}-q_{g}V_{m}}{q_{ion}L},$$

where *q_ion_* is the charge of the ion.

The barrier’s energy with respect to the ion’s position can be calculated through the following equation:

(5) $$U(x)=q_{ion}Ex,$$

Then, by substituting Equation (4) in Equation (5):

(6) $$U(x)=\frac{q_{g}V_{1/2}-q_{g}V_{m}}{L}x,$$

The channel’s gate is a short hydrophobic constriction that is located at the intracellular side [18,19]. Therefore, as long as the membrane potential is negative inside with regards to the outside, extracellular cations, such as sodium and potassium ions, will move from outside the cell using the membrane’s potential acquiring kinetic energy $q_{ion}V_{m}$ until hitting the intracellular gate [31,32]. On the other hand, the intracellular cations will hit the intracellular gate before going through the membrane’s potential and hence it will not affect their kinetic energy [31,32]. However, both intracellular and extracellular ions have a thermal energy at body temperature equals to $\frac{3}{2}K_{B}T=0.64\times 10^{-20}$ J. Therefore, it is assumed that the kinetic energy of the ion does not change while passing through the intracellular gate, since the length of the gate is relatively short when compared with the full thickness of the cell membrane; thus, the voltage across the gate is neglected.

The integral in Equation (3) can be solved as the following:

(7) $$R=\int_{X1}^{X2}\sqrt{\frac{q_{g}V_{1/2}-q_{g}V_{m}}{L}x-KE}dx=\frac{2L}{3(q_{g}V_{1/2}-q_{g}V_{m})}\sqrt{{\left(\frac{q_{g}V_{1/2}-q_{g}V_{m}}{L}x_{2}-KE\right)}^{3}}-\frac{2L}{3(q_{g}V_{1/2}-q_{g}V_{m})}\sqrt{{\left(\frac{q_{g}V_{1/2}-q_{g}V_{m}}{L}x_{1}-KE\right)}^{3}},$$

*x_2_* is at the end of the gate ($x_{2}=L$), and *x_1_* is where $U(x_{1})=\frac{q_{g}V_{1/2}-q_{g}V_{m}}{L}x_{1}=KE$. Thus, Equation (7) becomes:

(8) $$R=\int_{X1}^{X2}\sqrt{\frac{q_{g}V_{1/2}-q_{g}V_{m}}{L}x-KE}dx=\frac{2L}{3(q_{g}V_{1/2}-q_{g}V_{m})}\sqrt{{\left((q_{g}V_{1/2}-q_{g}V_{m})-KE\right)}^{3}},$$

Regarding the extracellular monovalent cations such as sodium and potassium, Equation (8) can be written as:

(9) $$R_{o}=\frac{2L}{-3(q_{g}V_{1/2}-q_{g}V_{m})}\sqrt{{\left(-(q_{g}V_{1/2}-q_{g}V_{m})-(q_{ion}V_{m}+\frac{3}{2}K_{B}T)\right)}^{3}},$$

(10) $$R_{o}=\frac{2L}{3(q_{g}V_{m}-q_{g}V_{1/2})}\sqrt{{\left((q_{g}V_{m}-q_{g}V_{1/2})-(q_{ion}V_{m}+\frac{3}{2}K_{B}T)\right)}^{3}},$$

In Equation (9), the minus sign is inserted to indicate that the membrane voltages (*V_m_* and *V*_1/2_) are absolute values. This is made because the kinetic energy of the ions $q_{ion}V_{m}$ is a positive value and *V_m_* should be an absolute value of the actual membrane voltage. Additionally, when tunneling probability and its related equations are encountered, the membrane’s voltage is negative on the inside with regard to the outside, and the value of the membrane’s voltage is an absolute value.

On the other hand, Equation (8) for the intracellular monovalent cations can be written as:

(11) $$R_{i}=\frac{2L}{3(q_{g}V_{m}-q_{g}V_{1/2})}\sqrt{{\left((q_{g}V_{m}-q_{g}V_{1/2})-(\frac{3}{2}K_{B}T)\right)}^{3}},$$

As a result, the tunneling probability of extracellular ions $T_{Q(o)}$ and intracellular ions $T_{Q(i)}$ can be calculated by the following equations:

(12) $$T_{Q(o)}=e^{-\frac{\sqrt{8m}}{\hbar }\times \frac{2L}{3(q_{g}V_{m}-q_{g}V_{1/2})}\sqrt{{\left((q_{g}V_{m}-q_{g}V_{1/2})-(q_{ion}V_{m}+\frac{3}{2}K_{B}T)\right)}^{3}}},$$

(13) $$T_{Q(i)}=e^{-\frac{\sqrt{8m}}{\hbar }\times \frac{2L}{3(q_{g}V_{m}-q_{g}V_{1/2})}\sqrt{{\left((q_{g}V_{m}-q_{g}V_{1/2})-(\frac{3}{2}K_{B}T)\right)}^{3}}},$$

So, from the perspective of quantum mechanics, ions can tunnel through the closed intracellular gate of the channels. Consequently, the closed channels can conduct ions via quantum tunneling, hence the conductance is called quantum conductance. The quantum conductance of a single channel $C_{Q}$ can be calculated by this equation [12,31,32,35]:

(14) $$C_{Q}=\frac{q^{2}{}_{ion}}{h}T_{Q},$$

where h is Planck’s constant ($6.6\times 10^{-34}$ Js), $q_{ion}$ is the charge of the ion, and $T_{Q}$ is the tunneling probability. The unit of $C_{Q}$ is Siemens (S).

Moreover, at certain channels’ density *D* (channels/m^2^), the quantum membrane conductance $C_{QM}$ can be calculated by this equation:

(15) $$C_{QM}=D\times C_{Q},$$

The unit of $C_{QM}$ is S/m^2^. 

## 3. Results

In this section, the comparison between quantum conductance and classical conductance of voltage-gated channels is made by graphing the equations, which contain the variables of conductance, gating free energy, and membrane voltage. Such presentation will delineate how the conductance changes alongside variations in gating free energy and membrane potential (voltage). The mathematical graphs are plotted using MATLAB. The mathematical graphing is used to provide a comprehensive evaluation of the electrical function of the voltage-gated channels. Moreover, by plotting the graphs and comparing them, novel perspectives relating to the voltage-gated channels and the contribution of the quantum behavior of ions in the pathophysiology of the excitability-related disorders could be discussed. The mathematical plots will be based on semi-log graphing so that the comparison between classical and quantum models can be made in a comprehensive manner. 

### 3.1. The Conductance of the Voltage-Gated Sodium Channels According to the Boltzmann Distribution 

In this study, the quantum and classical models will be applied on the Nav1.2 sodium channels. These channels have gating charge $q_{g}=9.2e=9.2\times 1.6\times 10^{-19}=14.72\times 10^{-19}$ C [36], and with half activation voltage $V_{1/2}=43$ mV [37,38], the gating free energy $q_{g}V_{1/2}=6.33\times 10^{-20}$ J [39]. Furthermore, the density of sodium channels D is $5\times 10^{13}$ channels/m^2^ [1], and the single channel conductance of sodium channel $C_{\sin gle(Na)}$ is $15\times 10^{-12}$ S [1].

Considering these values in Equations (1) and (2):

(16) $$P_{Na}={\left(1+e^{\frac{G-147.2V_{m}}{0.43}}\right)}^{-1},$$

(17) $$C_{M(Na)}=750\times {\left(1+e^{\frac{G-147.2V_{m}}{0.43}}\right)}^{-1},$$

where $G=\frac{q_{g}V_{1/2}}{10^{-20}}$. 

As long as the membrane potential is negative on the inside with regard to the outside, the membrane potentials (*V_m_* and *V*_1/2_) are substituted with their negative sign when the Boltzmann distribution is applied on the voltage-gated channels.

Thus, the open probability of sodium channels at G = −6.33 J:

(18) $$P_{Na}={\left(1+e^{\frac{-6.33-147.2V_{m}}{0.43}}\right)}^{-1},$$

**Figure 1:**
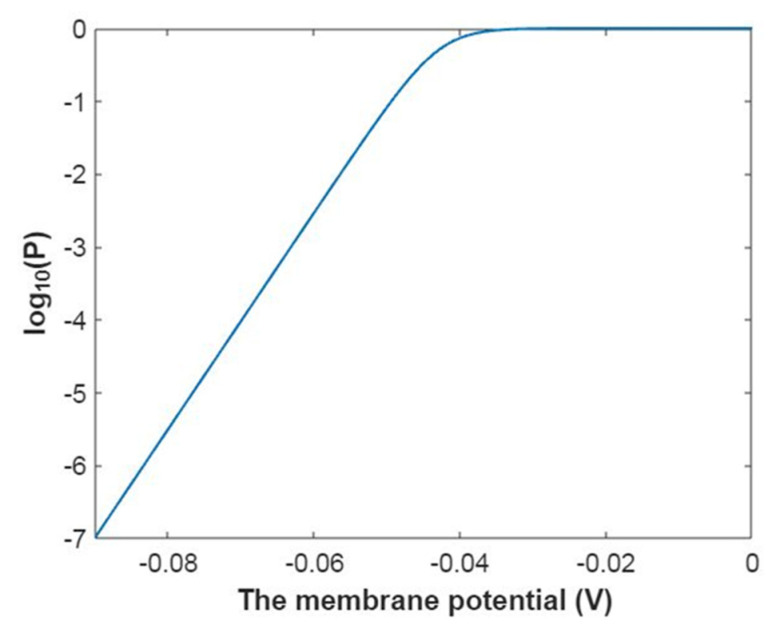
The relationship between the membrane potential and the common logarithm of the open probability of sodium channels at G = −6.33 J and over membrane potential range from −0.09 V to 0 V.

Then, the mathematical relationship between the membrane potential and the common logarithm of the open probability of the sodium channels can be plotted according to Equation (18) as in Figure 1.

The open probability of sodium channels at membrane potential −0.087 V:

(19) $$P_{Na}={\left(1+e^{\frac{G+12.81}{0.43}}\right)}^{-1},$$

**Figure 2:**
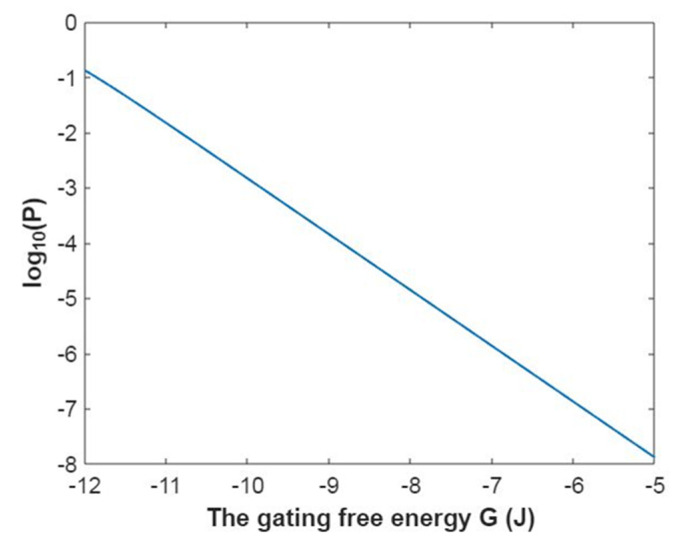
The relationship between gating free energy and the common logarithm of the open probability of the sodium channels at membrane potential −0.087 V and over gating free energy range from −12 J to −5 J.

Then, the mathematical relationship between gating free energy and the common logarithm of the open probability of the sodium channels can be plotted according to Equation (19) as in Figure 2. 

The reference point of membrane potential −0.087 V will be used throughout the paper to carry out the calculations where appropriate. This value of membrane potential represents the resting membrane potential at physiological concentrations and resting leaky conductance values of sodium and potassium ions (See Section 3.5 for values and calculations). 

The membrane conductance of sodium ions according to the Boltzmann distribution at G = −6.33 J:

(20) $$C_{M(Na)}=750\times {\left(1+e^{\frac{-6.33-147.2V_{m}}{0.43}}\right)}^{-1}$$

**Figure 3:**
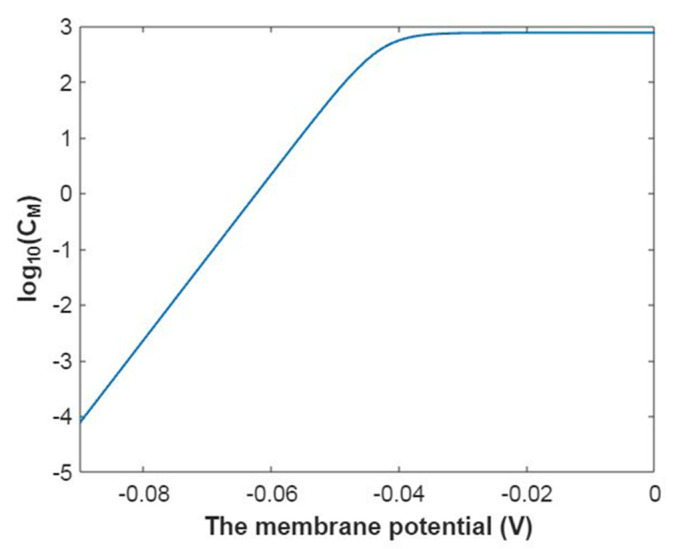
The relationship between membrane potential and the common logarithm of the membrane conductance of sodium ions according to the Boltzmann distribution at G = −6.33 J and over membrane potential range from −0.09 V to 0 V.

To evaluate how the membrane conductance changes with membrane potential, it is useful to mathematically graph the relationship between membrane potential and the common logarithm of the membrane conductance of sodium ions according to Equation (20) as in Figure 3.

On the other hand, the membrane conductance of sodium ions according to the Boltzmann distribution at membrane potential −0.087 V:

(21) $$C_{M(Na)}=750\times {\left(1+e^{\frac{G+12.81}{0.43}}\right)}^{-1},$$

**Figure 4:**
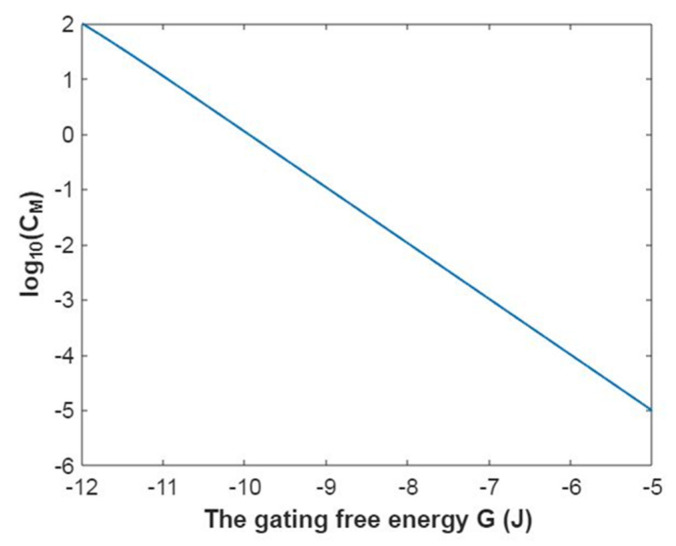
The relationship between gating free energy and the common logarithm of the membrane conductance of sodium ions according to the Boltzmann distribution at membrane potential −0.087 V and over gating free energy range from −12 J to −5 J.

In Figure 4, the mathematical graph of the relationship between gating free energy and the common logarithm of the membrane conductance of sodium ions is represented according to Equation (21). 

### 3.2. The Conductance of the Voltage-Gated Sodium Channels According to the Quantum Mechanics

#### 3.2.1. The Tunneling Probability of Sodium Ions through the Closed Intracellular Hydrophobic Gate

It is assumed that the intracellular hydrophobic gate has the length of a single hydrophobic residue within the alpha helix of the S6 segment and this length is $1.5\times 10^{-10}$ m [31,32,40] because the gate is formed when a single hydrophobic residue from each of the four S6 segments aggregates with other three hydrophobic residues [18,19]. Furthermore, in the previous papers [31,32,40], the length of the gate was calculated with consideration to the tilt angle of the S6 alpha helix with the plane of the membrane so that the length is reduced by the sine of this angle. However, in this work, the quantum-related graphs will be plotted at three different lengths $0.5\times 10^{-10}$ m, $1\times 10^{-10}$ m, and $1.5\times 10^{-10}$ m to show how the variables behave in reference to the gate’s length. Moreover, the mass of sodium ion is $3.8\times 10^{-26}$ Kg and its charge $q_{Na}=1e=1.6\times 10^{-19}$ will be used throughout the paper’s calculations. The model of quantum tunneling is applied on ions while passing through the hydrophobic gate of the voltage-gated channels and not the selectivity filter. Therefore, the hydrophobic nature of the gate promotes dehydrating the ions while tunneling through the gate [30]. Moreover, the quantum model is applied on the ‘closed’ gate conformation, which has narrower radius than the ‘open’ gate conformation. This narrow pore facilities ions dehydration [41]. Accordingly, in this study, the mass of ions (sodium and potassium ions) will be substituted as they are fully dehydrated due to hydrophobic nature of the gate and its narrow pore. 

Then, the tunneling probability of extracellular sodium ions can be calculated by the following equation:

(22) $$T_{Q}{}_{\left(Na(o)\right)}=e^{-\frac{35L\sqrt{{\left(131.2V_{m}-G-0.64\right)}^{3}}}{147.2V_{m}-G}},$$

while the tunneling probability of intracellular sodium ions can be calculated by the following equation:

(23) $$T_{Q}{}_{\left(Na(i)\right)}=e^{-\frac{35L\sqrt{{\left(147.2V_{m}-G-0.64\right)}^{3}}}{147.2V_{m}-G}},$$

All the ranges of membrane potential and gating free energy in the following plots will be chosen in a way by which their substitution in the aforementioned equations does not yield a negative number in the square root of the tunneling probability equation and its related equations of quantum conductance, in an effort to avoid getting imaginary numbers.

The tunneling probability of extracellular sodium ions through the energy barrier of the hydrophobic gate at G = 6.33 J:

(24) $$T_{Q(Na(o))}=e^{\frac{-35L\sqrt{{\left(131.2V_{m}-6.97\right)}^{3}}}{147.2V_{m}-6.33}},$$

**Figure 5:**
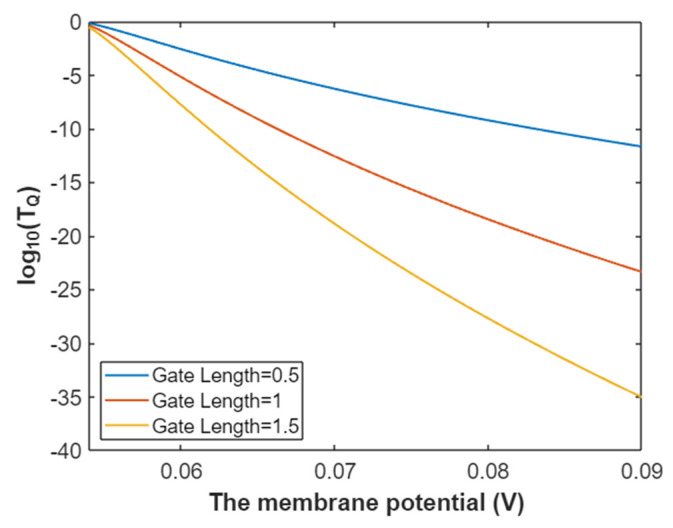
The relationship between the membrane potential and the common logarithm of tunneling probability of extracellular sodium ions at G = 6.33 J and over a membrane potential range from 0.054 V to 0.09 V.

The relationship between membrane potential and the common logarithm of tunnelling probability of extracellular sodium ions can be represented based on Equation (24) as in Figure 5.

The values of gating free energy and membrane potential in Equation (24) and Figure 5 are positive values because the membrane potentials (*V_m_* and *V*_1/2_) are substituted as the absolute values of their negative potentials. This is also will be valid wherever the quantum model is applied on the voltage-gated channels.

The tunneling probability of intracellular sodium ions through the energy barrier of the hydrophobic gate at G = 6.33 J:

(25) $$T_{Q(Na(i))}=e^{-\frac{35L\sqrt{{\left(147.2V_{m}-6.97\right)}^{3}}}{147.2V_{m}-6.33}},$$

**Figure 6:**
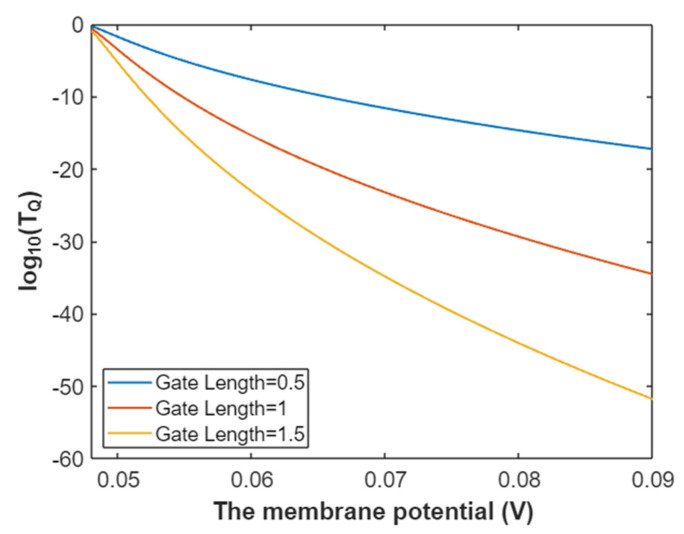
The relationship between membrane potential and the common logarithm of tunneling probability of intracellular sodium ions at G = 6.33 J and over a membrane potential range from 0.048 V to 0.09 V.

Then, the relationship between membrane potential and the common logarithm of tunneling probability of intracellular sodium ions can be graphed based on Equation (25) as in Figure 6. 

The tunneling probability of extracellular sodium ions at membrane potential 0.087 V:

(26) $$T_{Q(Na(o))}=e^{-\frac{35L\sqrt{{\left(10.77-G\right)}^{3}}}{12.81-G}},$$

**Figure 7:**
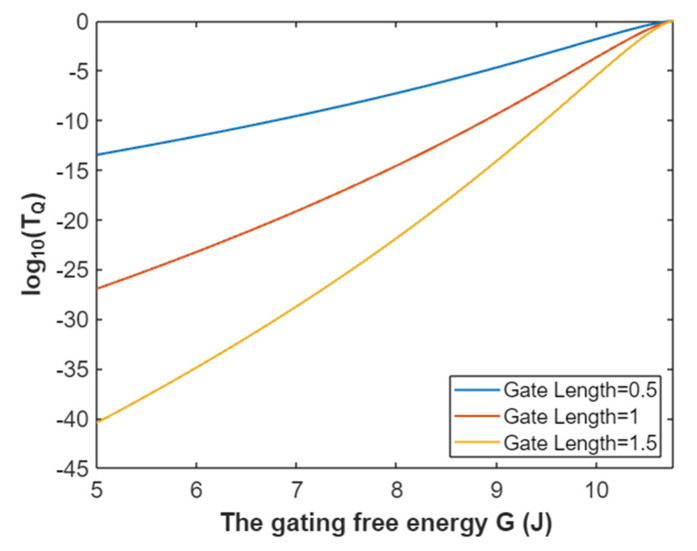
The relationship between gating free energy and the common logarithm of tunneling probability of extracellular sodium ions at membrane potential of 0.087 V and over a gating free energy range from 5 J to 10.77 J.

Accordingly, the relationship between gating free energy and the common logarithm of tunneling probability of extracellular sodium ions can be mathematically represented based on Equation (26) as in Figure 7.

The tunneling probability of intracellular sodium ions at membrane potential of 0.087 V:

(27) $$T_{Q(Na(i))}=e^{-\frac{35L\sqrt{{\left(12.17-G\right)}^{3}}}{12.81-G}},$$

**Figure 8:**
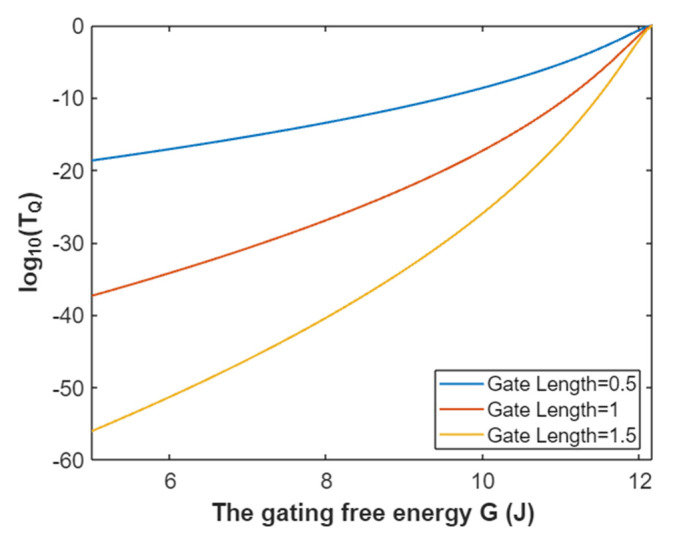
The relationship between gating free energy and the common logarithm of tunneling probability of intracellular sodium ions at membrane potential of 0.087 V and over a gating free energy range from 5 J to 12.17 J.

Hence, the mathematical graph of the relationship between the gating free energy and the common logarithm of tunneling probability of intracellular sodium ions can be plotted based on Equation (27) as in Figure 8. 

#### 3.2.2. The Quantum Conductance of Single Voltage-Gated Sodium Channel 

Substituting the available values in Equation (14), the quantum conductance of a single sodium channel for extracellular sodium ions can be calculated by the following equation:

(28) $$C_{QNa(o)}=3.88\times 10^{-5}\times e^{-\frac{35L\sqrt{{\left(131.2V_{m}-G-0.64\right)}^{3}}}{147.2V_{m}-G}},$$

On the other hand, the quantum conductance of a single sodium channel for intracellular sodium ions can be calculated by the following equation:

(29) $$C_{QNa(i)}=3.88\times 10^{-5}\times e^{-\frac{35L\sqrt{{\left(147.2V_{m}-G-0.64\right)}^{3}}}{147.2V_{m}-G}},$$

The quantum conductance of a single sodium channel at G = 6.33 J for extracellular sodium ions:

(30) $$C_{Q(Na(o))}=3.88\times 10^{-5}e^{-\frac{35L\sqrt{{\left(131.2V_{m}-6.97\right)}^{3}}}{147.2V_{m}-6.33}},$$

**Figure 9:**
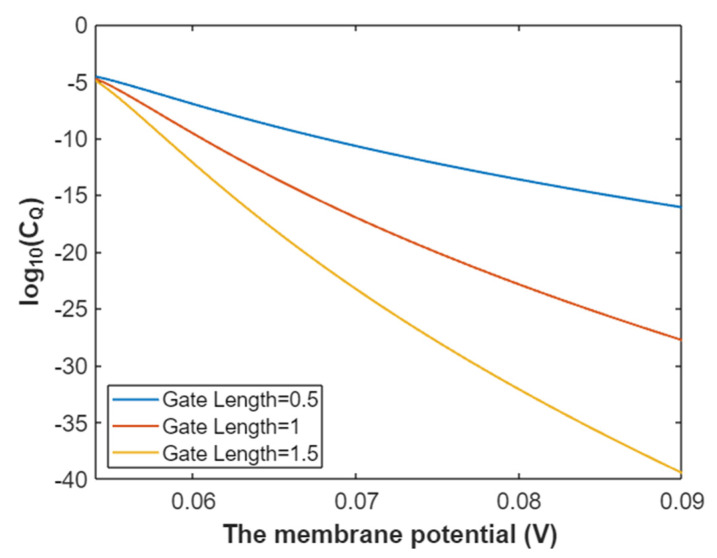
The relationship between membrane potential and the common logarithm of quantum conductance of a single sodium channel for extracellular sodium ions at G = 6.33 J and over a membrane potential range from 0.054 V to 0.09 V.

Then, according to Equation (30) the graph can be plotted to evaluate the relationship between membrane potential and the common logarithm of quantum conductance of a single sodium channel for extracellular sodium ions as in Figure 9.

The quantum conductance of a single sodium channel for intracellular sodium ions at G = 6.33 J:

(31) $$C_{Q(Na(i))}=3.88\times 10^{-5}e^{-\frac{35L\sqrt{{\left(147.2V_{m}-6.97\right)}^{3}}}{147.2V_{m}-6.33}},$$

**Figure 10:**
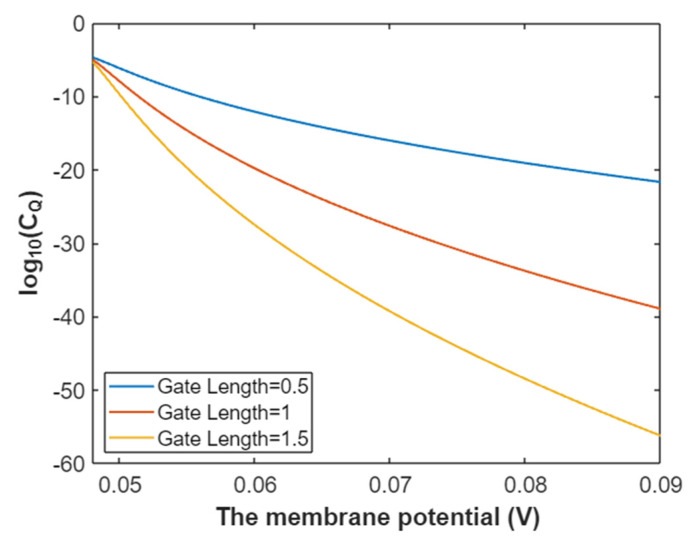
The relationship between membrane potential and the common logarithm of quantum conductance of a single sodium channel for the intracellular sodium ions at G = 6.33 J and over a membrane potential range from 0.048 V to 0.09 V.

In Figure 10, the graph of the relationship between membrane potential and the common logarithm of quantum conductance of a single sodium channel for intracellular sodium ions is represented according to Equation (31).

The quantum conductance of a single sodium channel for extracellular sodium ions at membrane potential 0.087 V:

(32) $$C_{Q(Na(o))}=3.88\times 10^{-5}e^{-\frac{35L\sqrt{{\left(10.77-G\right)}^{3}}}{12.81-G}},$$

**Figure 11:**
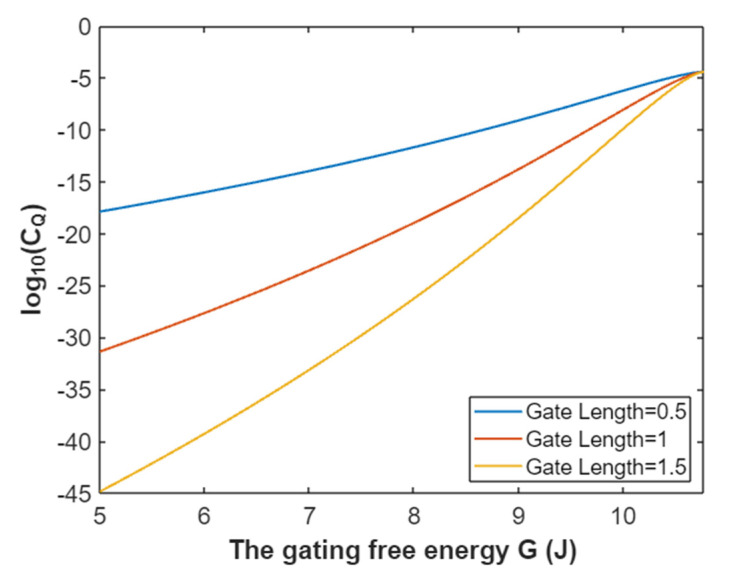
The relationship between gating free energy and the common logarithm of quantum conductance of a single sodium channel for the extracellular sodium ions at membrane potential 0.087 V and over a gating free energy range from 5 J to 10.77 J.

According to Equation (32), the graph that represents the relationship between the gating free energy and the common logarithm of quantum conductance of a single sodium channel for extracellular sodium ions is plotted as in Figure 11.

The quantum conductance of a single sodium channel for intracellular sodium ions at membrane potential 0.087 V:

(33) $$C_{Q(Na(i))}=3.88\times 10^{-5}e^{-\frac{35L\sqrt{{\left(12.17-G\right)}^{3}}}{12.81-G}},$$

**Figure 12:**
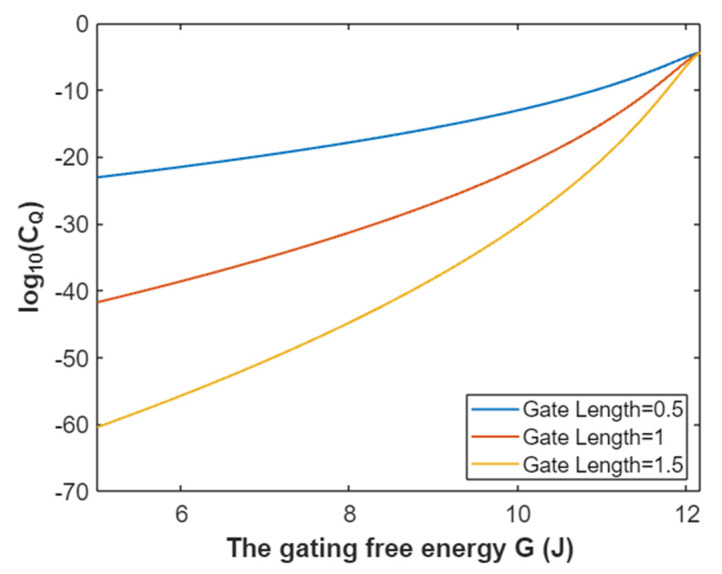
The relationship between gating free energy and the common logarithm of quantum conductance of a single channel for intracellular sodium ions at membrane potential 0.087 V and over a gating free energy range from 5 J to 12.17 J.

According to Equation (33), the relationship between gating free energy and the common logarithm of quantum conductance of a single sodium channel for the intracellular sodium ions can be graphed as in Figure 12. 

#### 3.2.3. The Quantum Membrane Conductance of Sodium Ions

Substituting the available values in Equation (15), the quantum membrane conductance of extracellular sodium ions can be calculated by the following equation:

(34) $$C_{QM(Na(o))}=1.94\times 10^{9}e^{-\frac{35L\sqrt{{\left(131.2V_{m}-G-0.64\right)}^{3}}}{147.2V_{m}-G}},$$

whereas the quantum membrane conductance of intracellular sodium ions can be calculated by the following equation:

(35) $$C_{QM(Na(i))}=1.94\times 10^{9}e^{\frac{-35L\sqrt{{\left(147.2V_{m}-G-0.64\right)}^{3}}}{147.2V_{m}-G}},$$

The unit of quantum membrane conductance $C_{QM}$ is S/m^2^.

The quantum membrane conductance of extracellular sodium ions at G = 6.33 J:

(36) $$C_{QM(Na(o))}=1.94\times 10^{9}e^{\frac{-35L\sqrt{{\left(131.2V_{m}-6.97\right)}^{3}}}{147.2V_{m}-6.33}},$$

**Figure 13:**
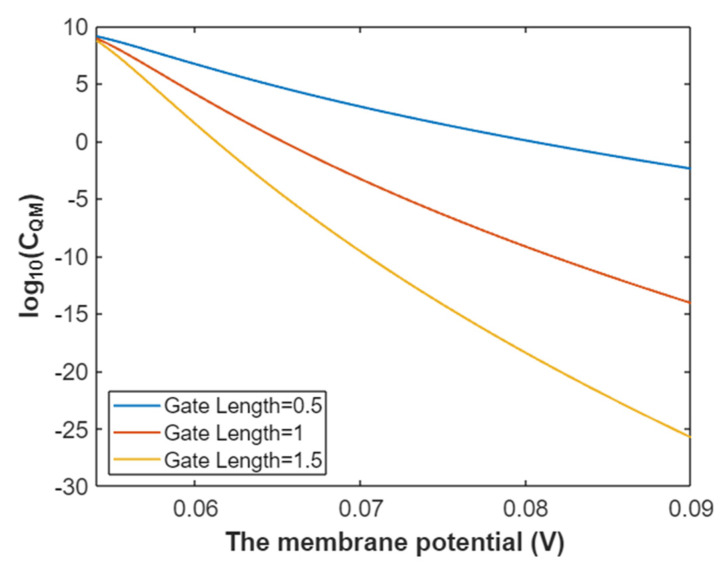
The relationship between membrane potential and the common logarithm of quantum membrane conductance of extracellular sodium ions at G = 6.33 J and over a membrane potential range from 0.054 V to 0.09 V.

Then, according to Equation (36) the relationship between membrane potential and the common logarithm of quantum membrane conductance of extracellular sodium ions can be mathematically graphed as in Figure 13.

The quantum membrane conductance of intracellular sodium ions at G = 6.33 J:

(37) $$C_{QM(Na(i))}=1.94\times 10^{9}e^{-\frac{35L\sqrt{{\left(147.2V_{m}-6.97\right)}^{3}}}{147.2V_{m}-6.33}},$$

**Figure 14:**
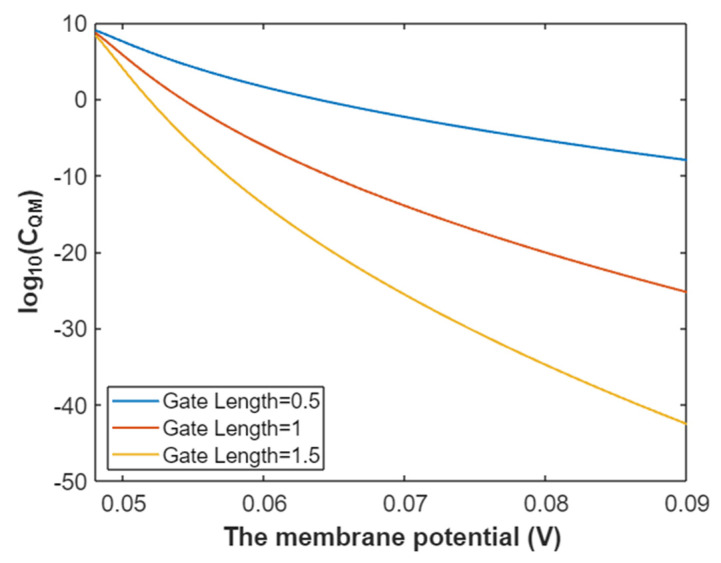
The relationship between membrane potential and the common logarithm of quantum membrane conductance of intracellular sodium ions at G = 6.33 J and over a membrane potential range from 0.048 V to 0.09 V.

According to Equation (37), the relationship between membrane potential and the common logarithm of quantum membrane conductance of intracellular sodium ions can be plotted as in Figure 14.

The quantum membrane conductance of extracellular sodium ions at membrane potential 0.087 V:

(38) $$C_{QM(Na(o))}=1.94\times 10^{9}e^{-\frac{35L\sqrt{{\left(10.77-G\right)}^{3}}}{12.81-G}},$$

**Figure 15:**
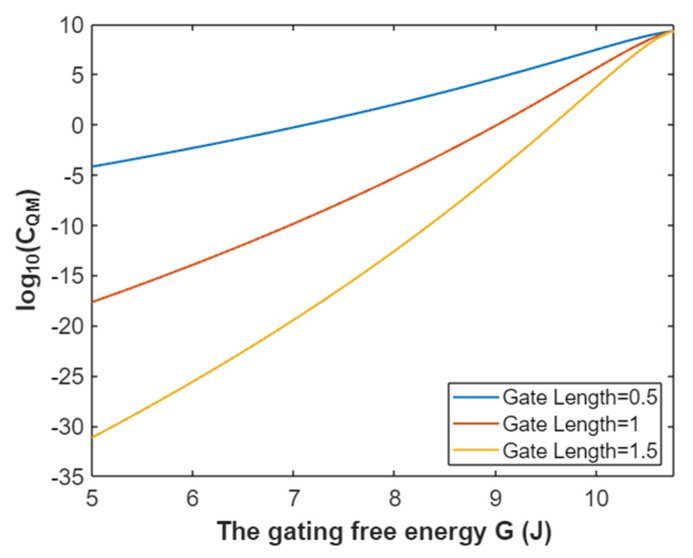
The relationship between gating free energy and the common logarithm of quantum membrane conductance of extracellular sodium ions at membrane potential of 0.087 V and over a gating free energy range from 5 J to 10.77 J.

Based on Equation (38), the relationship between gating free energy and the common logarithm of quantum membrane conductance of extracellular sodium ions can be mathematically represented as in Figure 15.

The quantum membrane conductance of intracellular sodium ions at membrane potential 0.087 V:

(39) $$C_{QM(Na(i))}=1.94\times 10^{9}e^{\frac{-35L\sqrt{{\left(12.17-G\right)}^{3}}}{12.81-G}},$$

**Figure 16:**
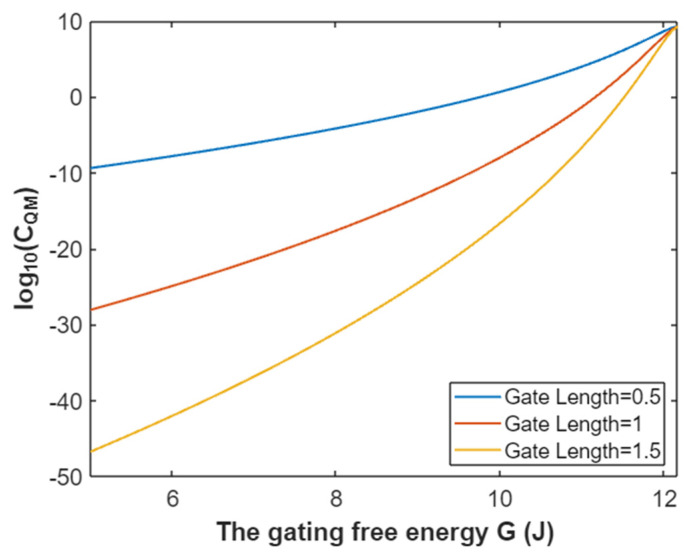
The relationship between gating free energy and the common logarithm of quantum membrane conductance of intracellular sodium ions at membrane potential of 0.087 V and over a gating free energy range from 5 J to 12.17 J.

Based on Equation (39), the mathematical representation of the relationship between gating free energy and the common logarithm of quantum membrane conductance of intracellular sodium ions is plotted as in Figure 16. 

### 3.3. The Conductance of the Voltage-Gated Potassium Channels According to the Boltzmann Distribution 

The models will be applied on the potassium channels K_V_1.2. These channels have gating charge $q_{g}=9.6e=15.36\times 10^{-19}$ C [42,43] and a gating free energy $q_{g}V_{1/2}=5.35\times 10^{-20}$ J [42,43]. Additionally, the density of potassium channels *D* will be substituted by $5\times 10^{13}$ channels/m^2^ [1] and the single channel conductance of potassium channel $C_{\sin gle(K)}=15\times 10^{-12}$ S [1]. 

The values of *D* and $C_{\sin gle}$ substituted for potassium channels are the same for the sodium channels, and this is reasonable because these channels could have the same parameters [1]. Furthermore, exact values are not the primary aim of the study but rather setting an applicable model in which different values can be substituted to predict the electrical behavior of ion channels. Furthermore, it is more suitable to set these parameters similarly so that a more reliable comparison can be made between sodium and potassium channels.

Considering these values in Equations (1) and (2):

(40) $$P_{K}={\left(1+e^{\frac{G-153.6V_{m}}{0.43}}\right)}^{-1},$$

(41) $$C_{M(K)}=750\times {\left(1+e^{\frac{G-153.6V_{m}}{0.43}}\right)}^{-1},$$

Thus, the open probability of potassium channels at G = −5.35 J:

(42) $$P_{K}={\left(1+e^{\frac{-5.35-153.6V_{m}}{0.43}}\right)}^{-1},$$

**Figure 17:**
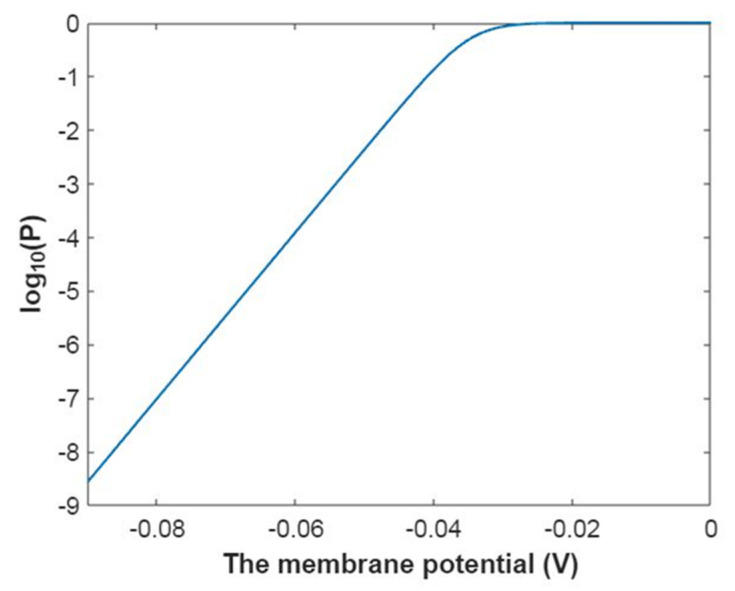
The relationship between the membrane potential and the common logarithm of the open probability of potassium channels at G = −5.35 J and over a membrane potential range from −0.09 V to 0 V.

Then, the relationship between membrane potential and the common logarithm of open probability of potassium channels can be plotted according to Equation (42) as in Figure 17.

Also, the open probability of potassium channels at membrane potential −0.087 V:

(43) $$P_{K}={\left(1+e^{\frac{G+13.36}{0.43}}\right)}^{-1},$$

**Figure 18:**
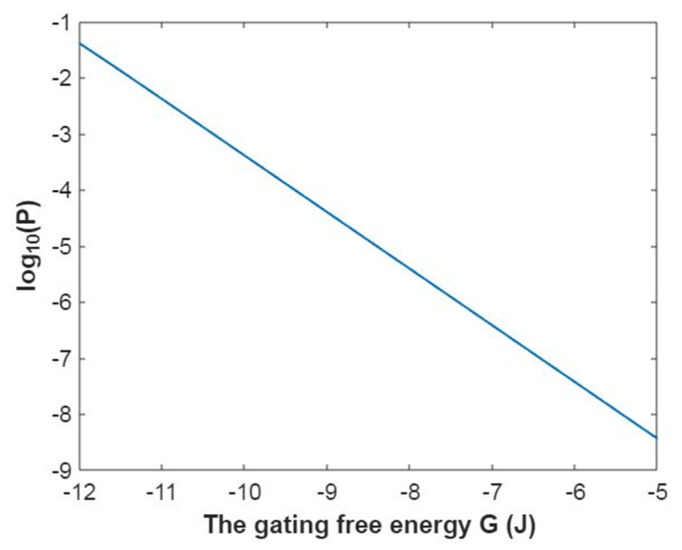
The relationship between gating free energy and the common logarithm of the open probability of potassium channels at membrane potential −0.087 V and over a gating free energy range from −12 J to −5 J.

Then, the relationship between gating free energy and the common logarithm of the open probability of potassium channels can be plotted according to Equation (43) as in Figure 18.

The membrane conductance of potassium ions at G = −5.35 J:

(44) $$C_{M(K)}=750\times {\left(1+e^{\frac{-5.35-153.6V_{m}}{0.43}}\right)}^{-1},$$

**Figure 19:**
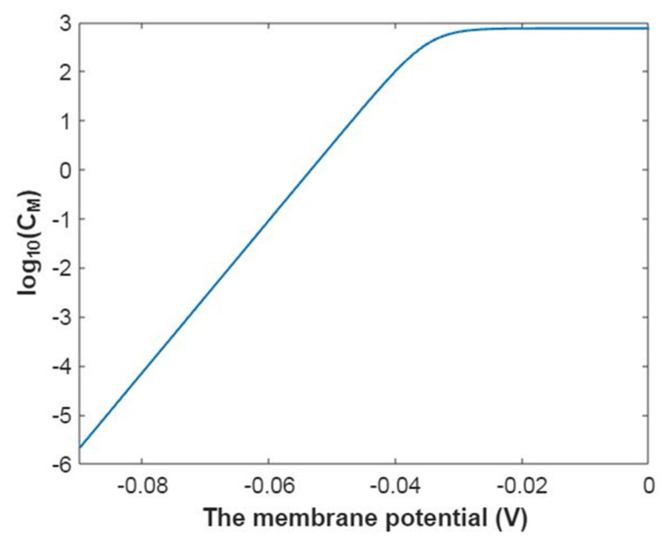
The relationship between membrane potential and the common logarithm of the membrane conductance of potassium ions according to the Boltzmann distribution at G = −5.35 J and over a membrane potential range from −0.09 V to 0 V.

The relationship between membrane potential and the common logarithm of the membrane conductance of potassium ions is plotted according to Equation (44) as in Figure 19. 

The membrane conductance of potassium ions at membrane potential −0.087 V:

(45) $$C_{M(K)}=750\times {\left(1+e^{\frac{G+13.36}{0.43}}\right)}^{-1},$$

**Figure 20:**
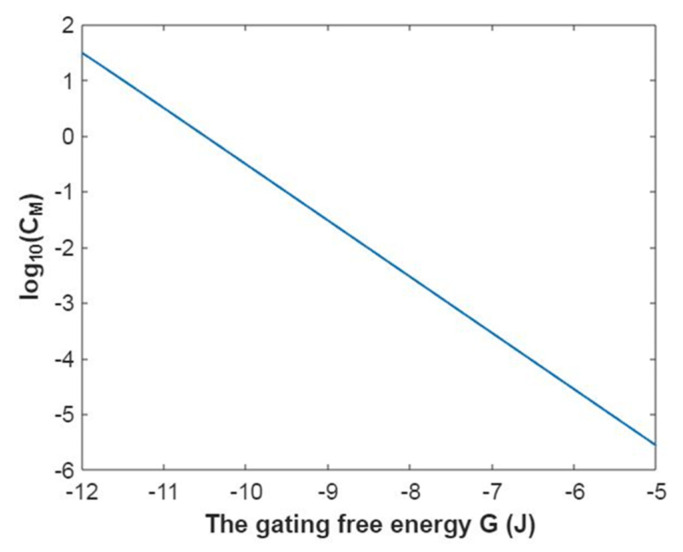
The relationship between gating free energy and the common logarithm of the membrane conductance of potassium ions according to the Boltzmann distribution at membrane potential of −0.087 V and over a gating free energy range from −12 J to −5 J.

Based on Equation (45), the relationship between gating free energy and the common logarithm of the membrane conductance of potassium ions can be mathematically graphed as in Figure 20.

### 3.4. The Conductance of the Voltage-Gated Potassium Channels According to the Quantum Mechanics

#### 3.4.1. The Tunneling Probability of Potassium Ions through the Intracellular Hydrophobic Gate

The intracellular hydrophobic gate in potassium channels is formed in a similar fashion to that of sodium channels [18,19]. Therefore, the quantum-related graphs for potassium channels will be plotted at three different lengths as were done in sodium channels: $0.5\times 10^{-10}$ m, $1\times 10^{-10}$ m, and $1.5\times 10^{-10}$ m. Furthermore, the mass of potassium ion is $6.5\times 10^{-26}$ Kg and its charge $q_{K}=1e=1.6\times 10^{-19}$ C. Accordingly, the tunneling probability of extracellular potassium ions can be calculated by the following equation:

(46) $$T_{Q(K(o))}=e^{\frac{-45.8L\sqrt{{\left(137.6V_{m}-G-0.64\right)}^{3}}}{153.6V_{m}-G}},$$

On the other hand, the tunneling probability of intracellular potassium ions can be calculated by the following equation:

(47) $$T_{Q(K(i))}=e^{\frac{-45.8L\sqrt{{\left(153.6V_{m}-G-0.64\right)}^{3}}}{153.6V_{m}-G}},$$

The tunneling probability of extracellular potassium ions at G = 5.35 J:

(48) $$T_{Q(K(o))}=e^{\frac{-45.8L\sqrt{{\left(137.6V_{m}-5.99\right)}^{3}}}{153.6V_{m}-5.35}},$$

**Figure 21:**
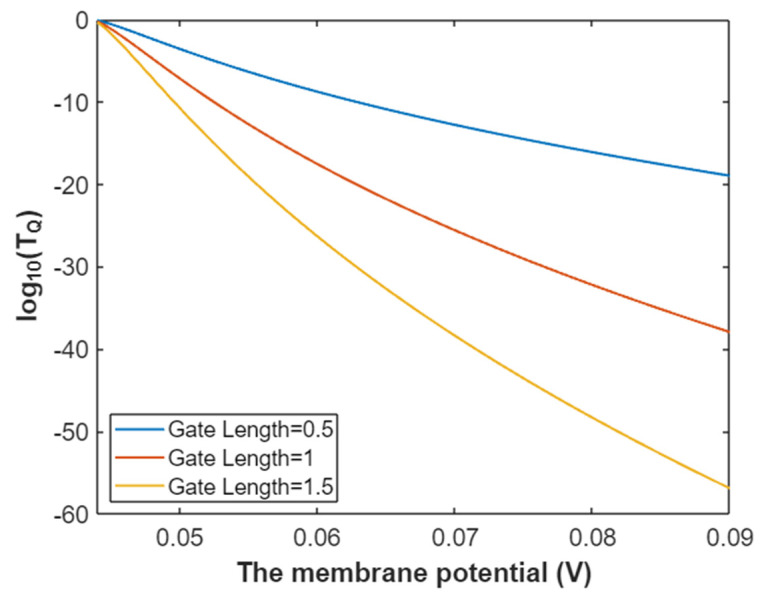
The relationship between membrane potential and the common logarithm of tunneling probability of extracellular potassium ions at G = 5.35 J and over a membrane potential range from 0.044 V to 0.09 V.

Based on Equation (48), the relationship between membrane potential and the common logarithm of tunneling probability of extracellular potassium ions can be represented as in Figure 21.

The tunneling probability of intracellular potassium ions at G = 5.35 J:

(49) $$T_{Q(K(i))}=e^{\frac{-45.8L\sqrt{{\left(153.6V_{m}-5.99\right)}^{3}}}{153.6V_{m}-5.35}},$$

**Figure 22:**
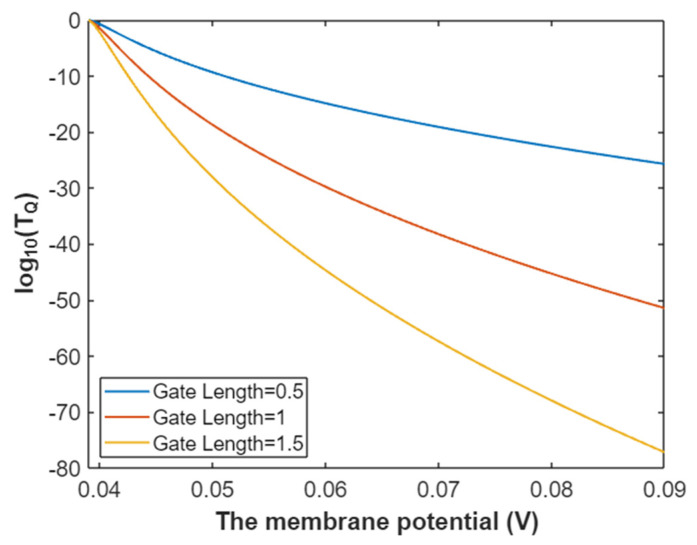
The relationship between membrane potential and the common logarithm of tunneling probability of intracellular potassium at G = 5.35 J and over a membrane potential range from 0.039 V to 0.09 V.

Based on Equation (49), the relationship between membrane potential and the common logarithm of tunneling probability of intracellular potassium ions can be mathematically graphed as in Figure 22.

The tunneling probability of extracellular potassium ions at membrane potential 0.087 V:

(50) $$T_{Q(K(o))}=e^{-\frac{45.8L\sqrt{{\left(11.33-G\right)}^{3}}}{13.36-G}},$$

**Figure 23:**
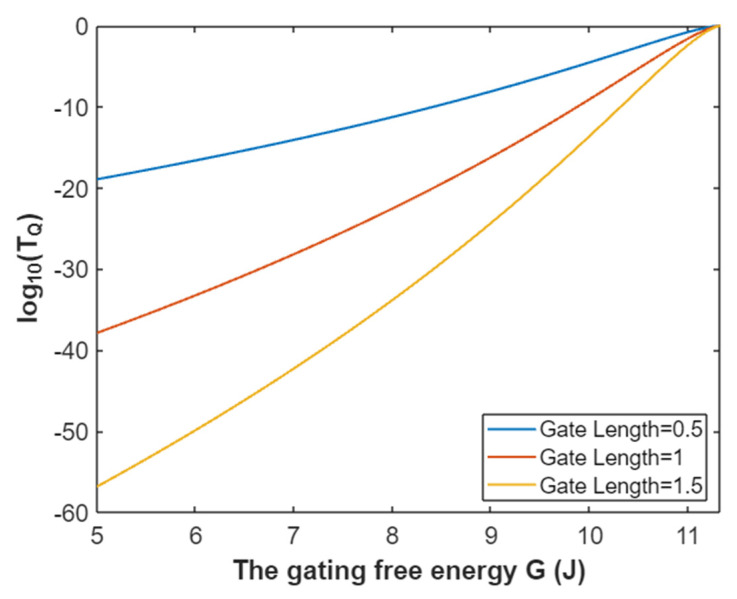
The relationship between gating free energy and the common logarithm of tunneling probability of extracellular potassium ions at membrane potential of 0.087 V and over a gating free energy range from 5 J to 11.33 J.

According to Equation (50), the relationship between gating free energy and the common logarithm of tunneling probability of extracellular potassium ions can be plotted as in Figure 23.

The tunneling probability of intracellular potassium ions at membrane potential 0.087 V:

(51) $$T_{Q(K(i))}=e^{-\frac{45.8L\sqrt{{\left(12.72-G\right)}^{3}}}{13.36-G}},$$

**Figure 24:**
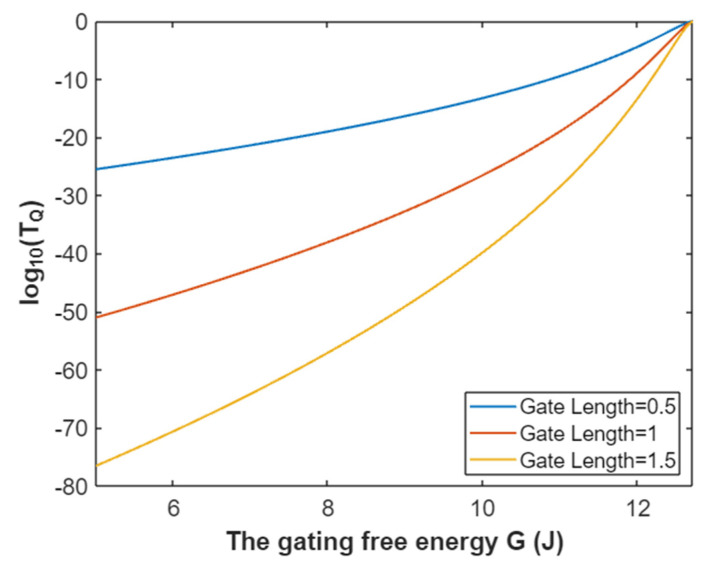
The relationship between gating free energy and the common logarithm of tunneling probability of intracellular potassium ions at membrane potential of 0.087 V and over a gating free energy range from 5 J to 12.72 J.

According to Equation (51), the relationship between gating free energy and the common logarithm of tunneling probability of intracellular potassium ions can be graphed as in Figure 24.

#### 3.4.2. The Quantum Conductance of Single Voltage-Gated Potassium Channel

The quantum conductance of single potassium channel for extracellular potassium ions at G = 5.35 J:

(52) $$C_{Q(K(o))}=3.88\times 10^{-5}e^{-\frac{45.8L\sqrt{{\left(137.6V_{m}-5.99\right)}^{3}}}{153.6V_{m}-5.35}},$$

**Figure 25:**
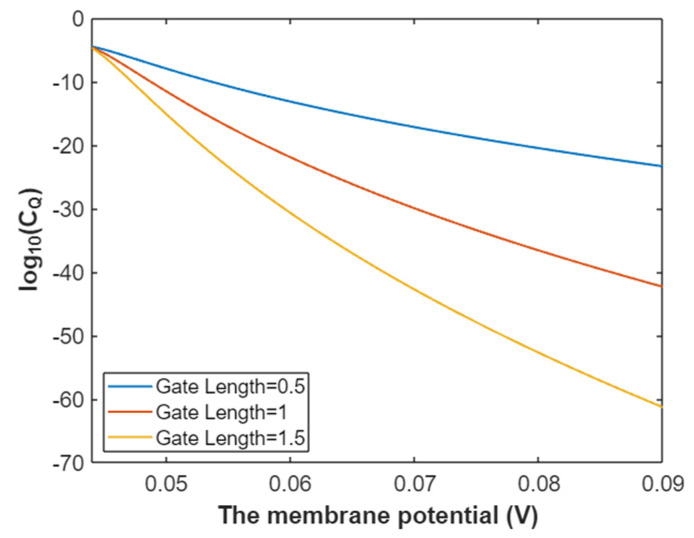
The relationship between membrane potential and the common logarithm of quantum conductance of a single potassium channel for extracellular potassium ions at G = 5.35 J and over a membrane potential range from 0.044 V to 0.09 V.

According to Equation (52), the relationship between membrane potential and the common logarithm of quantum conductance of a single potassium channel for extracellular potassium ions can be graphed as in Figure 25.

The quantum conductance of a single potassium channel for intracellular potassium ions at G = 5.35 J:

(53) $$C_{Q(K(i))}=3.88\times 10^{-5}e^{-\frac{45.8L\sqrt{{\left(153.6V_{m}-5.99\right)}^{3}}}{153.6V_{m}-5.35}},$$

**Figure 26:**
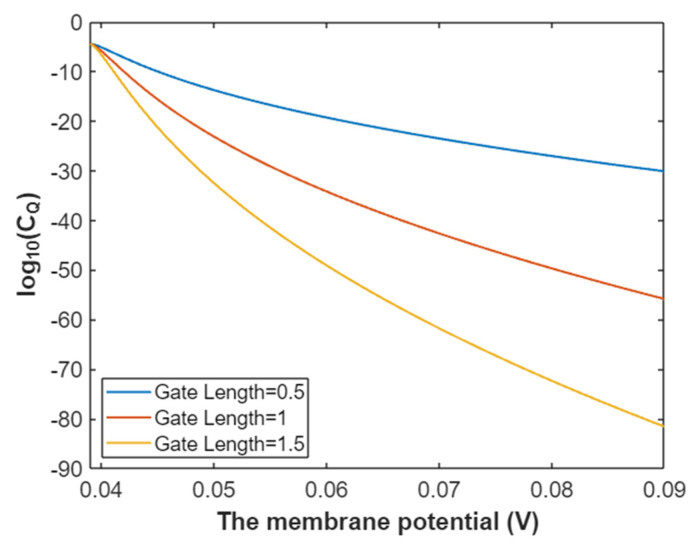
The relationship between membrane potential and the common logarithm of quantum conductance of a single potassium channel for intracellular potassium ions at G = 5.35 and over a membrane potential range from 0.039 V to 0.09 V.

According to Equation (53), the relationship between membrane potential and the common logarithm of quantum conductance of a single potassium channel for intracellular potassium ions can be graphed as in Figure 26.

The quantum conductance of a single potassium channel for extracellular potassium ions at membrane potential 0.087 V:

(54) $$C_{Q(K(o))}=3.88\times 10^{-5}e^{-\frac{45.8L\sqrt{{\left(11.33-G\right)}^{3}}}{13.36-G}},$$

**Figure 27:**
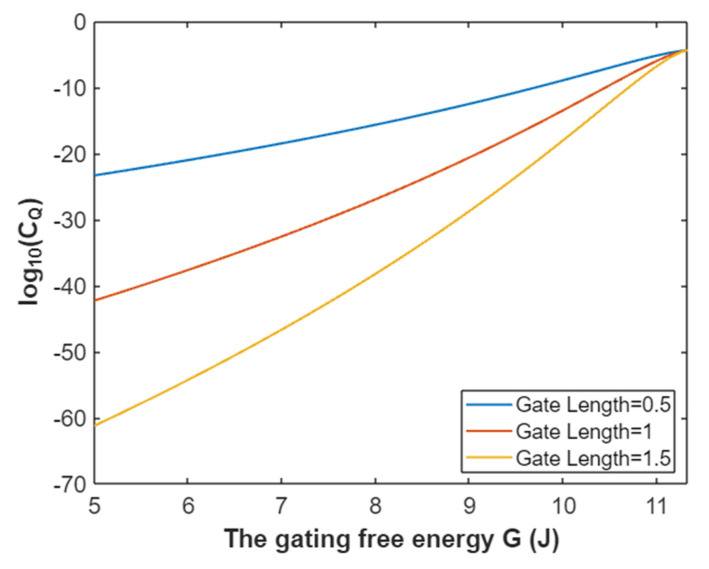
The relationship between gating free energy and the common logarithm of quantum conductance of a single potassium channel for extracellular potassium ions at membrane potential of 0.087 V and over a gating free energy range from 5 J to 11.33 J.

Based on Equation (54), the relationship between gating free energy and the common logarithm of quantum conductance of a single potassium channel for extracellular potassium ions can be mathematically represented as in Figure 27.

The quantum conductance of a single potassium channel for intracellular potassium ions at membrane potential 0.087 V:

(55) $$C_{Q(K(i))}=3.88\times 10^{-5}e^{-\frac{45.8L\sqrt{{\left(12.72-G\right)}^{3}}}{13.36-G}},$$

**Figure 28:**
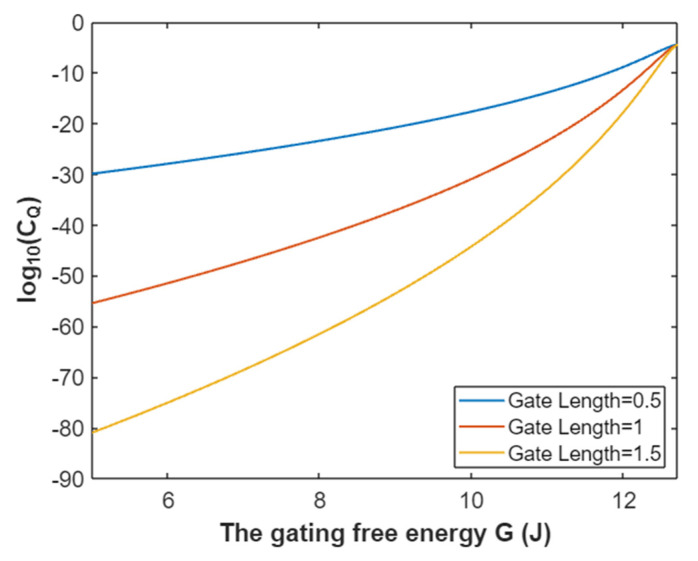
The relationship between gating free energy and the common logarithm of quantum conductance of a single potassium channel for intracellular potassium ions at membrane potential of 0.087 V and over a gating free energy range from 5 J to 12.72 J.

Based on Equation (55), the relationship between gating free energy and the common logarithm of quantum conductance of a single potassium channel for intracellular potassium ions can be mathematically represented as in Figure 28.

#### 3.4.3. The Quantum Membrane Conductance of Potassium Ions

Substituting the available values in Equation (15), the quantum membrane conductance of extracellular potassium ions can be calculated by the following equation:

(56) $$C_{QM(K(o))}=1.94\times 10^{9}e^{\frac{-45.8L\sqrt{{\left(137.6V_{m}-G-0.64\right)}^{3}}}{153.6V_{m}-G}},$$

On the other hand, the quantum membrane conductance of intracellular potassium ions can be calculated by the following equation:

(57) $$C_{QM(K(i))}=1.94\times 10^{9}e^{\frac{-45.8L\sqrt{{\left(153.6V_{m}-G-0.64\right)}^{3}}}{153.6V_{m}-G}},$$

The quantum membrane conductance of extracellular potassium ions at G = 5.35 J:

(58) $$C_{QM(K(o))}=1.94\times 10^{9}e^{\frac{-45.8L\sqrt{{\left(137.6V_{m}-5.99\right)}^{3}}}{153.6V_{m}-5.35}},$$

**Figure 29:**
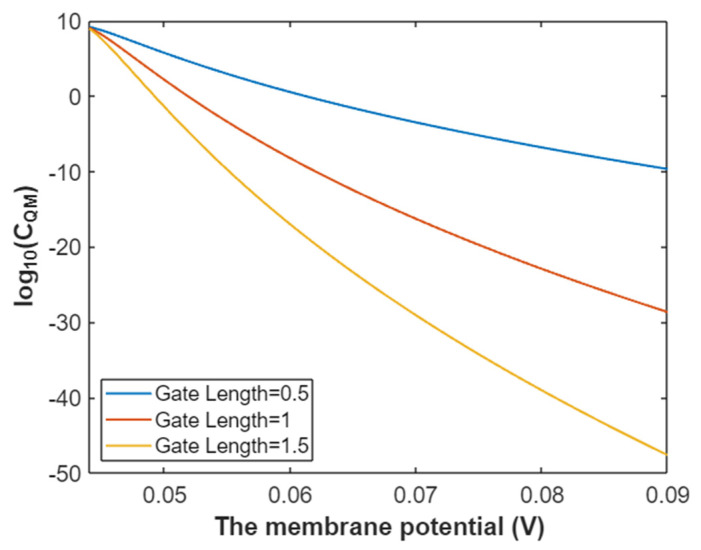
The relationship between membrane potential and the common logarithm of the quantum membrane conductance of extracellular potassium ions at G = 5.35 J and over a membrane potential range from 0.044 V to 0.09 V.

According to Equation (58), the relationship between membrane potential and the common logarithm of the quantum membrane conductance of extracellular potassium ions can be graphed as in Figure 29.

The quantum membrane conductance of intracellular potassium ions at G = 5.35 J:

(59) $$C_{QM(K(i))}=1.94\times 10^{9}e^{-\frac{45.8L\sqrt{{\left(153.6V_{m}-5.99\right)}^{3}}}{153.6V_{m}-5.35}},$$

**Figure 30:**
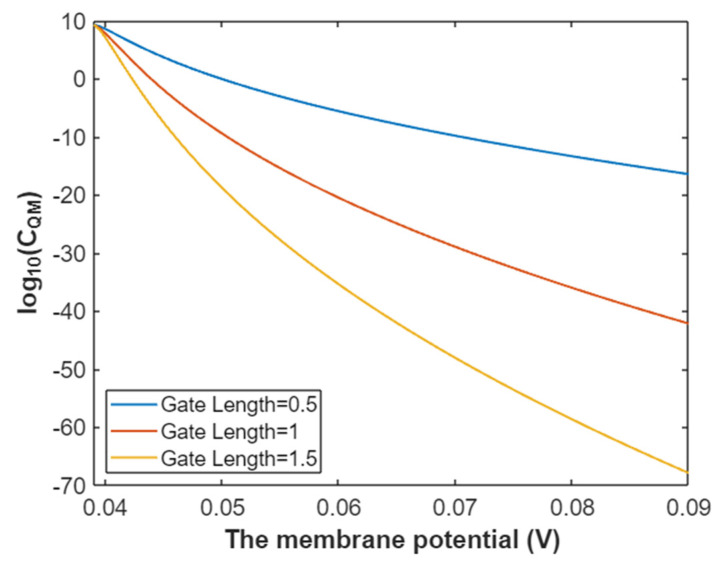
The relationship between membrane potential and the common logarithm of the quantum membrane conductance of intracellular potassium ions at G = 5.35 J and over a membrane potential range from 0.039 V to 0.09 V.

According to Equation (59), the relationship between membrane potential and the common logarithm of the quantum membrane conductance of intracellular potassium ions can be graphed as in Figure 30. 

The quantum membrane conductance of extracellular potassium ions at membrane potential 0.087 V:

(60) $$C_{QM(K(o))}=1.94\times 10^{9}e^{-\frac{45.8L\sqrt{{\left(11.33-G\right)}^{3}}}{13.36-G}},$$

**Figure 31:**
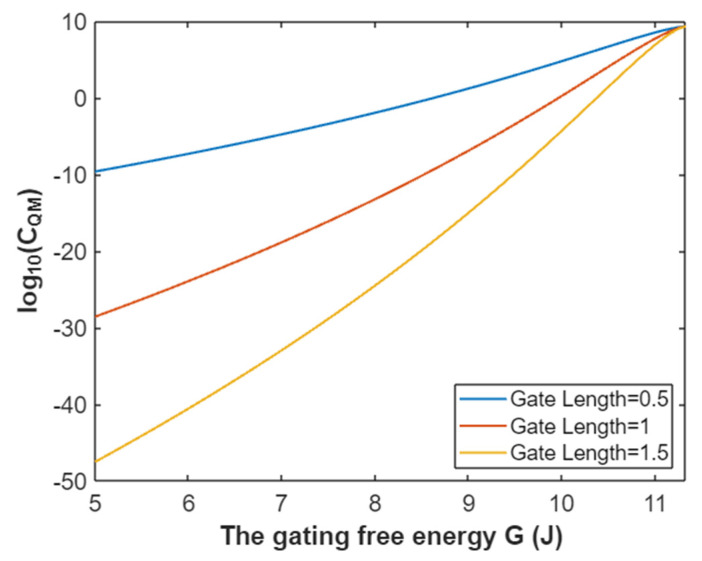
The relationship between gating free energy and the common logarithm of the quantum membrane conductance of extracellular potassium at membrane potential of 0.087 V and over a gating free energy range from 5 J to 11.33 J.

According to Equation (60), the relationship between gating free energy and the common logarithm of the quantum membrane conductance of extracellular potassium ions can be mathematically represented as in Figure 31. 

The quantum membrane conductance of intracellular potassium ions at membrane potential 0.087 V:

(61) $$C_{QM(K(i))}=1.94\times 10^{9}e^{-\frac{45.8L\sqrt{{\left(12.72-G\right)}^{3}}}{13.36-G}},$$

**Figure 32:**
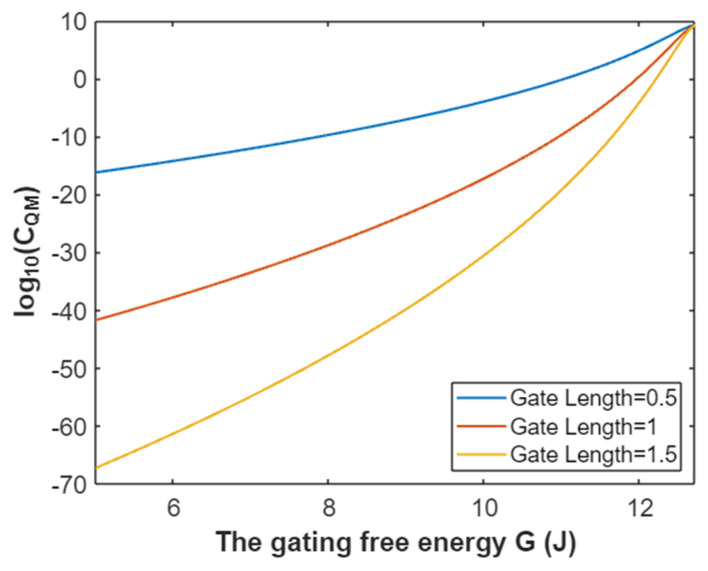
The relationship between gating free energy and the common logarithm of the quantum membrane conductance of intracellular potassium ions at membrane potential of 0.087 V and over a gating free energy range from 5 J and 12.72 J.

According to Equation (61), the relationship between gating free energy and the common logarithm of the quantum membrane conductance of intracellular potassium ions can be mathematically represented as in Figure 32.

### 3.5. The Influence of Quantum Tunneling of Ions on the Resting Membrane Potential

In the previous subsections, the focus was on the effect of gating free energy and membrane potential on the single channel conductance and membrane conductance from a quantum perspective. However, in this subsection, the focus is shifted on the effect of gating free energy on the resting membrane potential using the quantum version of the Goldman–Hodgkin–Katz equation. The resting membrane potential is an important electrical feature of excitable tissues because it determines the degree of excitability and response of such tissues to stimulus [44]. Thus, addressing resting membrane potential using a quantum model is useful to assess the influence of the quantum behavior of ions on excitability.

According to the classical version of the Goldman–Hodgkin–Katz equation, the resting membrane potential is determined when the net flux of ions across the membrane is zero. The flux of ions is driven by two gradients: (1) The chemical gradient that drives the flux according to the differences in ion concentration, and (2) the electrical gradient that drives the flux according to the differences in voltage. Therefore, when these two gradients are balanced and the net flux is zero, the membrane potential is determined under an electrochemical equilibrium. However, a third gradient can be added if the quantum aspect of ions is considered, which is the quantum gradient. The quantum gradient is generated because the extracellular cations acquire higher kinetic energy as they move through the membrane’s voltage; hence they have higher tunneling probability and higher conductance than that of intracellular cations [32]. Thus, cations will pass from the outside to the inside of a cell down their quantum gradient via quantum tunneling. 

Consequently, the Goldman–Hodgkin–Katz equation integrating the quantum conductance of ions can be written as the following [32]:

(62) $${\left[Na\right]}_{o}\left(C_{Na}+C_{QM(Na(o))}\right)+{\left[K\right]}_{o}\left(C_{K}+C_{QM(K(o))}\right)=e^{\frac{FV_{m}}{RT}}\left({\left[Na\right]}_{i}(C_{Na}+C_{QM(Na(i))})+{\left[K\right]}_{i}(C_{K}+C_{QM(K(i))})\right),$$

where (*o*) means extracellular, (*i*) means intracellular, [ ] means concentration, $C_{Na}$ is the membrane conductance of sodium ions at the resting state due to leaky channels (0.05 S/m^2^) [1,45], $C_{K}$ is the membrane conductance of potassium ions at the resting state due to leaky channels (5 S/m^2^) [1,45], *F* is Faraday’s constant (96,485.33 C/mol), *R* is the gas constant (8.31 J/Kmol), *T* is the body temperature (310 K), and *V_m_* is the resting membrane potential at the equilibrium. As it was explained before, the membrane potential *V_m_* is an absolute value of the negative potential of the membrane. Furthermore, the following values will be considered for the ions concentrations: ${\left[Na\right]}_{o}=142$ mmol/L [45], ${\left[Na\right]}_{i}=14$ mmol/L [45], ${\left[K\right]}_{o}=4$ mmol/L [45], and ${\left[K\right]}_{i}=140$ mmol/L [45]. 

By substituting the above values of leaky membrane conductance and concentrations of sodium and potassium in Equation (62) and by ignoring the quantum conductance of ions (the classical version of GHK equation), the resting membrane potential $V_{m}=0.087$ V. 

The quantum gradient of cations tends to depolarize the resting membrane potential because extracellular cations have higher conductance than their intracellular counterparts which indicates that there is more flux of positive charges to the inside of a cell. 

The activation gate, where the quantum model is applied, operates classically by dilating its pore in response to membrane depolarization to facilitate the conduction of ions. On the other hand, another opposing conformational change will happen to counteract the conduction of ions in response to membrane depolarization. This opposing conformational change is the formation of the inactivation gate that blocks the conduction of ions [1]. Even though that both the activation and inactivation gates are formed in response to membrane depolarization, the activation gate has faster kinetics than the inactivation gate and this allows the conduction of ions [1]. This is important as it facilitated the formulation of action potential signals in excitable tissues [1].

On the other hand, both the activation and the inactivation events of the voltage-gated channels would occur in a case of equilibrium because it is slower process than the action potential generation [1]. Therefore, the membrane depolarization at the equilibrium will cause the voltage-gated channel to undergo the conformational changes of activation and inactivation. Considering the equilibrium, the intracellular hydrophobic constriction will not depend on the membrane voltage to shape its energy barrier. Additionally, this consequence seems consistent and reasonable especially when the inactivation gate is formed at the same intracellular hydrophobic constriction where the activation gate is formed [46]. Therefore, it is assumed that the conformational changes of the inactivation event will balance the conformational changes of the activation event preserving the original barrier’s parameters of length and gating energy. This assumption is made to make it easier to study the influence of quantum tunneling of ions on the resting membrane potential under the equilibrium. Consequently, this makes the barrier energy of the hydrophobic gate independent of the membrane potential. 

Therefore, we modified the equation of tunneling probability for extracellular sodium ions at membrane potential of 0.087 V as the following:

(63) $$T_{Q(Na(o))r}=e^{-\frac{35L\sqrt{{\left(12.17-G-16V_{m}\right)}^{3}}}{12.81-G}},$$

also, the equation of tunneling probability for extracellular potassium ions at membrane potential of 0.087 V can be modified as the following:

(64) $$T_{Q(K(o))r}=e^{-\frac{45.8L\sqrt{{\left(12.72-G-16V_{m}\right)}^{3}}}{13.36-G}},$$

Based on Equations (63) and (64), the value of membrane potential in this expression $q_{g}V_{m}$ as in Equation (12) is substituted by 0.087 V because the intracellular hydrophobic constriction is balanced by two opposite events (activation and inactivation). The activation dilates the pore while inactivation constricts the pore. Thus, it is assumed that the net effect of these two events is no change in the radius of the pore; hence the hydrophobic constriction does not depend on the membrane voltage changes to shape its energy barrier. Accordingly, the original resting membrane potential, which is 0.087 V, is substituted in this expression $q_{g}V_{m}$. On the other hand, the membrane potential in the kinetic energy of the ion $16V_{m}$ is kept as a variable because the ion can still be influenced by the membrane potential regardless of the state of activation or inactivation. 

Even though there are other suggested mechanisms of inactivation [47,48], our case focuses on the net effect of conformational changes of activation and inactivation, which resembles the original barrier parameters in terms of length and energy. This implies that ions can tunnel through the inactivation gate by the same principles applied on the activation gate.

By substituting the available values in Equation (62) and taking into consideration the quantum conductance of sodium ions:

(65) $$27.1+2.76\times 10^{11}e^{-\frac{35L\sqrt{{\left(12.17-G-16V_{m}\right)}^{3}}}{12.81-G}}=700.7e^{-37.45V_{m}},$$

The quantum membrane conductance of intracellular sodium ions is neglected because it is much lower than the quantum membrane conductance of extracellular sodium ions.

**Figure 33:**
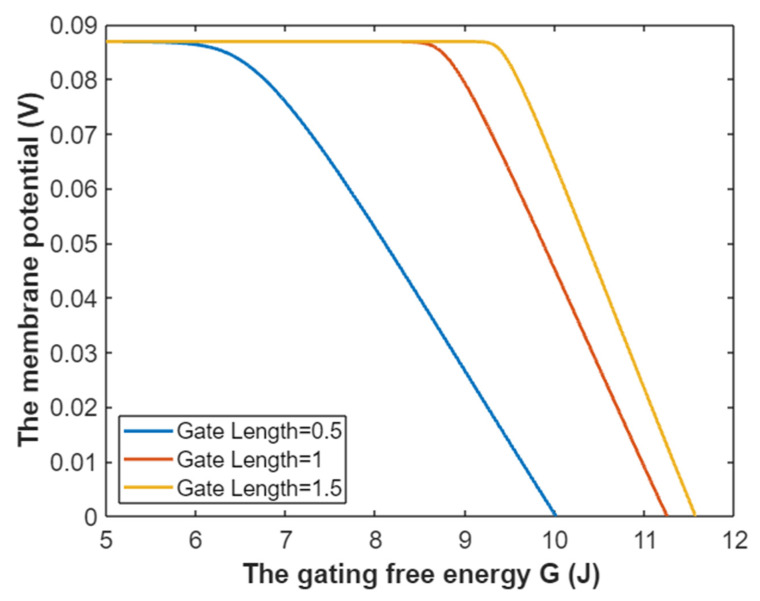
The relationship between gating free energy and the resting membrane potential under the influence of quantum tunneling of sodium ions.

To evaluate the influence of quantum tunneling of sodium ions on the resting membrane potential, Equation (65) is used to graph the relationship between gating free energy and resting membrane potential as in Figure 33.

By substituting the available values in Equation (62) and taking into consideration the quantum conductance of potassium ions:

(66) $$27.1+7.76\times 10^{9}e^{-\frac{45.8L\sqrt{{\left(12.72-G-16V_{m}\right)}^{3}}}{13.36-G}}=700.7e^{-37.45V_{m}},$$

The quantum conductance of intracellular potassium ions is neglected because it is much lower than the quantum conductance of extracellular potassium ions.

**Figure 34:**
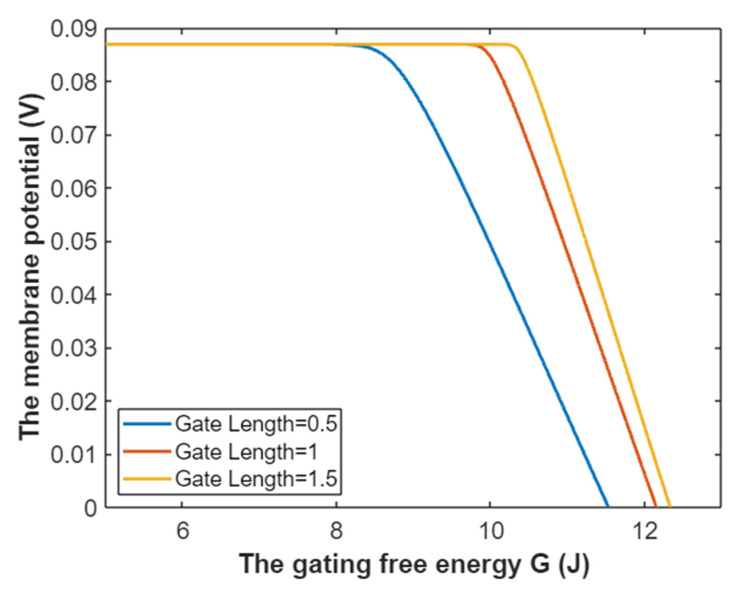
The relationship between gating free energy and the resting membrane potential under the influence of quantum tunneling of potassium ions.

To evaluate the influence of quantum tunneling of potassium ions on the resting membrane potential, Equation (66) is used to graph the relationship between the gating free energy and the resting membrane potential as in Figure 34.

## 4. Discussion

In this section, the function of the voltage-gated channels will be discussed according to plotted graphs demonstrated in the results. Additionally, a comparison will be made between the quantum model and the classical model of Boltzmann distribution. Moreover, the contributions of the quantum behavior of ions in the pathophysiology of certain diseases and disorders of the excitable tissues will be elaborated upon. 

**Figure 35:**
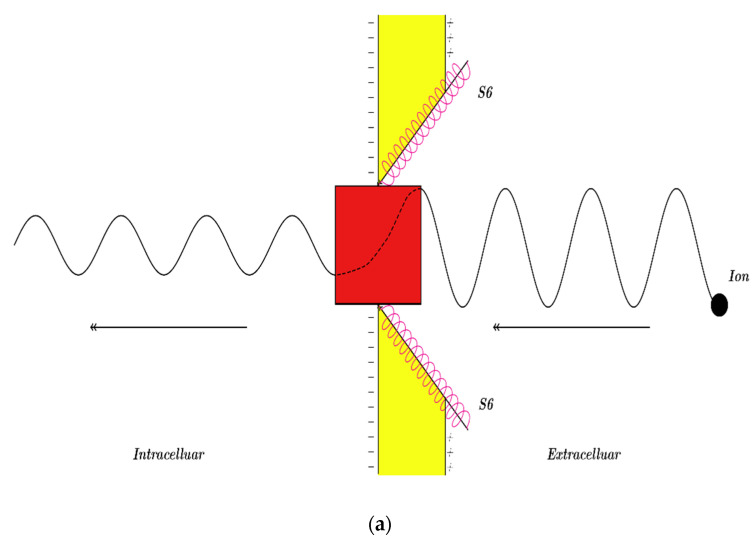

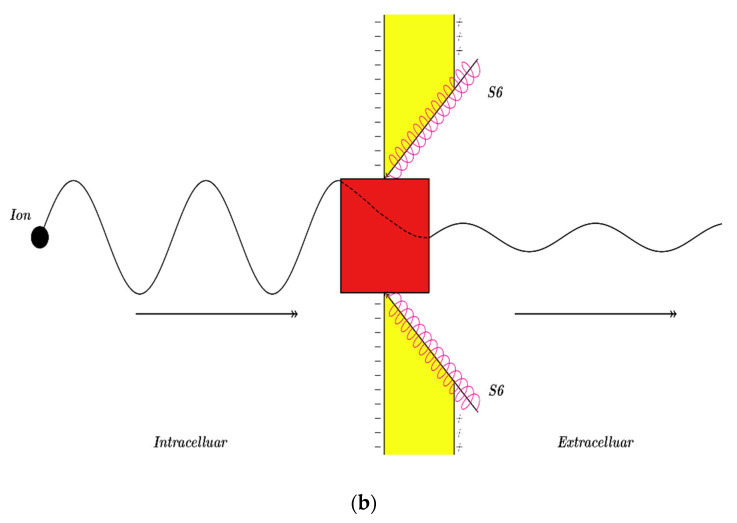
A schematic diagram represents the quantum wave behavior of an ion and the quantum tunneling through the intracellular gate (shown in red) indicated by the dashed line in the gate, which is formed by the crossing of the four S6 segments near the intracellular side (two of them are shown for simplicity). Figure (**a**) represents quantum tunneling of an ion moving from the extracellular side to the intracellular side. Figure (**b**) represents quantum tunneling of an ion moving from the intracellular side to the extracellular side. Notice that the extracellular ion, as in Figure (**a**), has shorter wavelength (the distance from peak to peak) when it is compared with that of the intracellular ion, as in Figure (**b**). This indicates that extracellular ions have higher kinetic energy since it is inversely correlated with the wavelength. Additionally, the wave amplitude of the extracellular ion passing after the gate, as in Figure (**a**), is higher than that of the intracellular ion, as in Figure (**b**). This indicates that extracellular ions have higher tunneling probability since it is proportional to the wave amplitude.

To make the discussion well-organized, the results are interpreted in a numerical fashion:

1. The opening of the gate is mediated by the movement of the four voltage sensors, which in turn dilates the hydrophobic constriction in order to conduct ions [49]. This opening needs a certain level of energy to be achieved. The classical model of Boltzmann distribution calculates the fraction of the total channels that has the enough required energy to open the channels for the conduction of ions. On the other hand, the quantum model is based on the quantum tunneling of ions through the intracellular hydrophobic gate. Quantum tunneling implies that ions possess a non-zero probability to pass through the gate even when the gate’s energy requirement is higher than that of the ions themselves. So, the quantum tunneling of ions through a channel is the origin of that channel’s quantum conductance. Additionally, the tunneling probability depends exponentially on the mass of an ion, the length of the gate, the gating free energy, and the kinetic energy of an ion. 

2. According to the quantum model, a voltage-gated channel has a spectrum of single channel conductance values and not just two states as described in the classical model: (1) Open, which conducts ions and (2) closed, which does not conduct ions. So, even though that the channel appears to be structurally closed, it is still able to conduct ions by quantum tunneling. Moreover, the classical model states that an ion can pass through the channel when the energy of the hydrophobic constriction drops to a certain level at which the ion has enough energy to overcome the barrier energy. This drop in the energy happens when the hydrophobic pore dilates, thus increasing its radius. On the other hand, the quantum model offers the possibility of ion passage through the closed hydrophobic gate even though that the energy of the barrier is higher than the energy of the ion. This is an important quantum property of voltage-gated channels. This observation renders the two classical states of voltage-channels obsolete in determining the conductance of a single channel. This provides a reasonable explanation for the permeation of ions through the closed channels without the need of the mechanical movement of voltage sensors to dilate the pore because quantum tunneling of ions does not require the pore dilation [50,51]. Moreover, the quantum model can explain that the movement of at least one voltage sensor out of four, can make a channel permeable [52]. Such phenomenon exists due to the movement of one voltage sensor as it can partially dilate the pore, reducing the barrier energy of the gate to enhance the quantum tunneling of ions and their conductance without the need of the cooperation of the four sensors to fully dilate the pore. 

3. Based on the equations and graphs in the results section, there is a probability of a channel opening in the classical model and there is a probability of ion tunneling through the channel in the quantum model. In addition to that, there are two values of single channel conductance in the classical model: (1) Zero conductance when the channel is closed and (2) $C_{\sin gle}$ when the channel is open, but there is a range of single channel conductance values in the quantum model and not just two values. However, there is membrane conductance in both models.

4. As aforementioned in the results, the tunneling probability, quantum conductance of a single channel, and quantum membrane conductance were evaluated according to the membrane potential and gating free energy. It is obvious that the extracellular cations have higher tunneling probability, higher single channel conductance, and higher membrane conductance because they have higher kinetic energy than their intracellular counterparts. The higher kinetic energy of the extracellular cations is attributed to the membrane potential that is negative on the inside in comparison to the outside of a cell; hence the cations that come from the outside passing through the channel until reaching the intracellular gate will go through the membrane potential and acquire kinetic energy. Therefore, the quantum model discriminates between the extracellular and intracellular ions in terms of conductance unlike the classical model, which treats them the same in terms of conductance; see Figure 35.

Additionally, it is clear that sodium has higher tunneling probability and higher conductance than potassium and this discrepancy can be attributed to the fact that the mass of sodium is less than the mass of potassium. Thus, the mass is an exponentially influential factor on the conductance in the quantum model.

5. The membrane conductance is the eventual electrical property that is used to assess the influence of ions on the excitability of the membrane. By comparing the graphs of membrane conductance for the same ion in both models, it is evident that the quantum membrane conductance of extracellular ions can be comparable or even higher than the membrane conductance of ions due to the mechanical opening of the channels as in the classical model. This means that ions can achieve significant quantum conductance, which can affect the electrical behavior of excitable cells at the resting state and during an action potential.

6. The membrane potential affects quantum tunneling and the conductance of ions in both models. This is vital in the generation and propagation of action potentials because if a stimulus is strong enough, it will trigger the opening of the sodium voltage-gated channels to increase their conductance, depolarizing the membrane potential, which will further increase the conductance of sodium ions to elicit a spike of depolarization. According to the graphs in the results, it seems that quantum tunneling of ions contributed to the depolarization spike of the action potential because as the membrane depolarizes, the quantum membrane conductance of sodium ions increases significantly in comparison to the membrane conductance in the Boltzmann model. Thus, this insight gives a new perspective in the process of action potential generation involving the quantum behavior of ions. Furthermore, this observation supports the previous works that focused on the role of quantum mechanics within action potentials [53,54]. 

7. Based on the graphs in the results, it is apparent that the gating free energy affected the membrane conductance. The gating free energy is the energy required to open the closed channel at membrane potential 0 V and this quantity gives information about how difficult it is to open a channel. Additionally, the conductance was assessed over a decreasing range of gating free energy; hence it was expected to observe increasing values in the conductance. The gating free energy of the channels decreases in many conditions including: Channelopathies [55,56,57] tissue ischemia [58], and mechanical stretch of the membrane [58,59]. These conditions are present in many genetic and acquired diseases such as channelopathies of epilepsy [60], cardiac arrhythmias [61], and pain disorders [62]. Moreover, cerebral ischemia, stroke, myocardial ischemia, and myocardial infarction are associated with increased risk of higher excitability resulting in seizures, epilepsy, and life-threatening arrhythmias [63,64,65]. Furthermore, the pathological stretch in heart failure and valvular heart disease is associated with serious arrhythmias such as ventricular tachycardia, ventricular fibrillation, and atrial fibrillation [66,67,68,69]. So, the decrease in the gating free energy can increase the risk of developing excitability-related disorders. This can be explained by the two models: (1) In the classical model, the decrease in the gating free energy of the sodium channels will decrease the barrier energy of the gate resulting in a higher fraction of open channels and higher membrane conductance at certain membrane voltage. This means that the membrane initiates the depolarization spike of action potential at lower thresholds in response to certain stimulus. (2) In the quantum model, the decrease in the gating free energy of the sodium channels will decrease the barrier energy of the gate resulting in higher tunneling probability and higher quantum conductance of sodium ions at certain membrane voltages. Consequently, the membrane will be more responsive and more able to generate the depolarization spike of action potentials at a lower threshold. Furthermore, according to the quantum model, the decrease in the gating free energy results in a significant increase in the conductance of extracellular sodium ions that can affect the membrane potential and its excitability. Thus, the decrease in the gating free energy will decrease the threshold required to initiate an action potential according to both models. Interestingly, there is another mechanism through which the quantum behavior of ions can increase this excitability. This mechanism is the ability of sodium and potassium ions to depolarize the resting membrane potential in response to the decrease in the gating free energy as represented in Figure 33 and Figure 34. Therefore, as the resting membrane potential becomes depolarized, it becomes closer to the threshold and more responsive to transmit an action potential.

8. According to the quantum model, both sodium and potassium ions can depolarize the resting membrane potential because extracellular sodium and potassium ions have higher tunneling probability and higher quantum conductance than intracellular sodium and potassium ions. Consequently, there will be a quantum gradient of positive ions from outside to inside the cell resulting in depolarizing the resting membrane potential under the equilibrium. However, according to the classical model, when the potassium channels open, potassium ions diffuse from inside to outside the cell, down their concentration gradient, resulting in membrane hyperpolarization. Hence, the quantum model can explain why gain-of-function mutations in potassium channels of excitatory neurons can result in hyperexcitability instead of hypoexcitability [70]. Surprisingly, when the gain-of-function mutation in potassium channels decreases the gating free energy, this may increase the tunneling probability and quantum conductance of extracellular potassium ions significantly to depolarize the resting membrane potential, predisposing the membrane to hyperexcitability. 

9. The quantum model treats voltage-gated channels as leaky channels that can contribute to the resting membrane potential especially when certain mutation occurs to decrease the gating free energy. On the other hand, the classical model concerns itself with the opening and closing of channels for initiation and propagation of action potentials. Under equilibrium, the ions can tunnel through the activation or the inactivation gate resulting in a leaky persistent quantum current that could alter the membrane potential. This serves to give a reasonable mechanism behind the persistent inward sodium current in the mutated channels, which are implicated in higher excitability such as in epilepsy. This current fails to be inactivated even during prolonged depolarization [71] and this is consistent with the prediction of the quantum model, which states that ions can tunnel through the inactivation gate during even prolonged depolarization. 

The comparisons and the implications listed above are deduced mainly from the equations and the mathematical graphs obtained in the results. Therefore, they require experimental evidence to be further validated. However, it is vital to propose and discuss the mathematical structure of any model before performing any experiments to test its validity. Hence, the present study focuses on the mathematical aspects of the quantum bioelectrical property of the biological membrane.

To make a connection between the mathematical quantum model proposed here in the article and future experiments to test its validity, concrete strategies and approaches are listed here to help in conducting experimental studies aimed at providing evidence on the quantum bioelectrical property of the biological membrane. Interestingly, it has been shown that the quantum features of ions can be used to control the membrane preparation which shows a high selectivity for targeted ions [72]. 

Before addressing the strategies and the approaches, it is important to notice that under normal physiological parameters the quantum behavior of sodium and potassium ions has minimal influence on the overall electrical behavior of the excitable cells [31,32]. This is clear in Figure 33 and Figure 34 in which the gating free energy must decrease to certain level at which the quantum behavior has a significant effect on the membrane potential. Therefore, when electrophysiological studies are to be conducted, it is important to set an experimental environment where the quantum behavior of ions can influence the membrane potential. This can be achieved by two main approaches: (1) Inducing mutations in the residues of the activation or inactivation gates so that the gating free energy decreases or (2) using ions with lower masses such as lithium [31,32] or hydrogen ions (protons) so that the quantum tunneling probability increases significantly without even inducing mutations in the activation or inactivation gates. 

The evidence for the quantum bioelectrical property of the biological membrane can be provided by adopting the following experimental strategies and approaches:

1. A key difference between classical mechanics and quantum mechanics of the voltage-gated channels is the process of ions conduction as aforementioned. According to quantum mechanics, ions can tunnel through the closed activation gate or inactivation gate without inducing any conformational changes in the molecular structure of the voltage-gated channel such as the movements of the voltage sensors or pore dilations. This can give a promising hint on the presence of quantum effects of ions. Thus, electrophysiological studies can be conducted to measure such quantum currents while observing no mechanical movements or pore dilation sufficient to conduct the ions.

2. According to quantum mechanics, the closed channels can have a range of single channel conductance values. Hence, it is expected that different molecular closed structures of the same mutated channel would have different values of conductance that obey the exponential equations presented here in the article. Therefore, electrophysiological studies can be conducted to measure the conductance of the different closed structures of the same channel before reaching the open state in which sufficient dilation has occurred to permeate ions. Then, the experimental results of the conductance values can be compared with the theoretical results obtained when applying the mathematical model proposed in the present work. Another similar approach is to perform different mutations on the same channel to decrease the gating free energy. Hence, the conductance at a certain closed structure is measured experimentally for the different mutations performed. Then the experimental and the theoretical values are compared to evaluate the validity of the mathematical quantum model. In addition, according to the quantum model, the quantum conductance of a single channel can be significantly larger than the closed state of a channel. Thus, experimentally manipulating the parameters of the closed hydrophobic gate can result in conductance values higher than the values of the closed state and this can provide evidence on quantum conductance. 

3. One important quantum aspect in the function of the voltage-gated channel is that extracellular cations have higher conductance than the intracellular cations and this is applied on both sodium and potassium ions. For example, when a gain-of-function mutation (mutation decreases the gating free energy) occurs in the potassium channel, it is expected that there will be a net inward (from the extracellular side to the intracellular side) current of potassium ions. Therefore, electrophysiological studies can be performed to measure this inward current of potassium ions through the mutated channels. This will provide promising evidence on the presence of the quantum effects because this inward current of potassium ions cannot be explained by the conventional electrophysiological principles as potassium ions must diffuse according to their concertation gradient from the intracellular side to the extracellular side resulting in only an outward current. 

4. Here, another approach is proposed to be utilized to provide the evidence on the quantum effects of the biological membrane. The approach is to measure the resting membrane potential at different groups of mutated channels contained in the biological membrane. Then, it is expected to get experimental plots that fit the theoretical plots as in Figure 33 and Figure 34. Additionally, observing the depolarizing effect of potassium ions when the gating free energy decreases due to mutations will provide favorable evidence for the effects of quantum behavior within biological systems. This effect is not expected according to the classical mechanics or the conventional electrophysiological principles. Actually, according to classical mechanics, it is not expected for the voltage-gated channels to contribute to establishing the resting membrane potential because they mainly function during the action potential propagation and not at the resting state. However, according to quantum mechanics, it is expected for the mutated voltage-gated channels to contribute to establishing the resting membrane potential as discussed before. Therefore, if experiments provide evidence on the relationship between the gating free energy of the voltage-gated channels and the resting membrane potential that are consistent with the theoretical data proposed here, this will provide evidence that the biological membrane possesses quantum electrical activity. 

5. Furthermore, a key feature in the quantum conductance is the exponential dependence on the mass of the ions and the length of the gate. This will be an interesting strategy to be used to experimentally show the exponential variations in quantum conductance between ions according to their masses and the length of the gate. 

Quantum de-coherence is one of the fastest physical processes and it represents a major challenge for the validity of many studies that focus on applying quantum mechanics on the biological systems including our present study. We mentioned this challenge in the introduction. Here, we provide possible mechanisms that can sustain the quantum coherence of ions so that the quantum tunneling can occur before de-coherence takes place. These mechanisms include:

1. The short length of the hydrophobic gate $(0.5-1.5)\times 10^{-10}$ m, in which the quantum tunneling happens, will provide enough time before de-coherence occurs because it has been shown that the distance correlates inversely to the time of de-coherence [17,73,74].
2. The gate is made by the aggregation of hydrophobic amino acids. This hydrophobic pocket will exclude water (dewetting) and may create a decoherence-free subspace [17,75].
3. Because voltage-gated channels and ions are a part of the biological system, it is expected that they could operate far from the thermal equilibrium, which indicates that ions may cool down through the hydrophobic gate even at higher body temperatures. This ‘cooling down’ can sustain the quantum coherence and make the quantum effects more apparent [17,74,75,76,77].
4. The complex non-linearity of brain dynamics can augment the quantum fluctuations and hence contribute to the maintenance of the quantum coherence of ions [78,79]. The electromagnetic field effects possibly mediate this augmentation [80,81,82].

The present work has certain qualities that make it distinct from the other previous studies [31,32]. These qualities include: (1) The present study makes a comparison between classical mechanics and quantum mechanics to mathematically show the differences in their influence on the function of voltage-gated channels. (2) The present study integrates the effect of membrane potential on the energy barrier of the gate. Thus, this allows us to evaluate the relationship between membrane potential and quantum tunneling probability, quantum conductance of a single channel, and the quantum membrane conductance. (3) The present study uses mathematical graphs to show vividly how the aforementioned variables change and behave according to the principles of quantum mechanics. Using mathematical graphs allows covering a wide range of values to get a more comprehensive understanding of the quantum model of the voltage-gated channels. (4) The present study focuses on both the activation and inactivation gate in its discussion. (5) The present study sheds light on the implications of using the quantum model to provide reasonable explanations for the pathological mechanisms in excitability-related diseases and disorders. (6) The present study sets a more comprehensive mathematical model that can be used to measure to the quantum conductance of the channels and the biological membrane in electrophysiological experiments.

## 5. Conclusions

The present study proposes the quantum model, which is based on the quantum tunneling of ions through the closed gate of these channels, to approach voltage-gated channels. This approach is adopted to unveil new properties in the voltage-gated channels and to solve the challenges and problems at the molecular and clinical levels. The model can explain the permeation of ions through the closed channels without the movement of voltage sensors, and it can also explain the paradox of the hyperexcitability effects of gain-of-function mutations in potassium channels. Moreover, it can explain the persistent inward sodium current in the mutated channels during prolonged depolarization. In addition to that, the quantum model sheds light on the contribution of the quantum tunneling of ions on the pathophysiology of epilepsy, pain, and arrhythmia. This might be helpful to develop new agents to treat and control these chronic and challenging diseases. Also, different experimental strategies are listed in this study to test the scientific validity of the proposed model. Furthermore, possible mechanisms are suggested to explain the persistence of quantum coherence of ions to ensure that quantum tunneling of ions occurs before de-coherence.

## Data Availability

Not applicable.
